# Effects of *Ficus carica* L. Leaf Extract on Ipsilateral and Contralateral Testes After Testicular Torsion/Detorsion Injury: Evaluation of Oxidative Stress, Inflammation, and Apoptosis

**DOI:** 10.3390/ijms27198678

**Published:** 2026-09-28

**Authors:** Zeynep Yığman, Saadet Özen Akarca Dizakar, Metin Yığman, Mürşide Ayşe Demirel, İpek Süntar, Ali Doğan Dursun, Ayhan İbrahim Aysal, Ayşe Köylü, Suna Ömeroğlu

**Affiliations:** 1 Department of Histology and Embryology, Faculty of Medicine, Gazi University, 06500 Ankara, Türkiye; 2 Neuroscience and Neurotechnology Center of Excellence (NÖROM), Gazi University, 06560 Ankara, Türkiye; 3 Department of Histology and Embryology, Faculty of Medicine, Bakırçay University, 35665 İzmir, Türkiye; 4 Urology Division, Ministry of Health Etlik City Hospital, 06170 Ankara, Türkiye; 5 Department of Basic Pharmaceutical Sciences, Faculty of Pharmacy, Gazi University, 06330 Ankara, Türkiye; aysedemirel@gazi.edu.tr; 6 Department of Pharmacognosy, Faculty of Pharmacy, Gazi University, 06330 Ankara, Türkiye; ipesin@gazi.edu.tr; 7 Center for Laboratory Animal Breeding and Experimental Research (ATUDEM), Atilim University, 06830 Ankara, Türkiye; 8 Vocational School of Health Services, Atilim University, 06830 Ankara, Türkiye; 9 Home Care Services, Medicana International Ankara Hospital, 06510 Ankara, Türkiye; 10 Department of Analytical Chemistry, Faculty of Pharmacy, Gazi University, 06330 Ankara, Türkiye; 11 Department of Histology and Embryology, Faculty of Medicine, Istinye University 34475 İstanbul, Türkiye; ayskoylu@gmail.com

**Keywords:** testicular torsion/detorsion, *F. carica* L. leaf extract, ipsilateral testis, contralateral testis, oxidative stress, apoptosis, inflammation, rat

## Abstract

The current study aimed to investigate the dose-dependent effects of *Ficus carica* leaf extract on oxidative, inflammatory, and apoptotic processes contributing to ischemia–reperfusion injury in bilateral testes following testicular torsion–detorsion. Forty-eight male Wistar Albino rats were assigned into six groups (*n* *=* 8): Intact, Sham, Torsion/Detorsion (T/D), T/D + 125, T/D + 250, and T/D + 500. Sixty minutes of left testicular torsion was followed by 56-day reperfusion in all T/D, T/D + 125, T/D + 250, and T/D + 500 groups, and rats in these groups were daily administered with vehicle, 125 mg/kg, 250 mg/kg, and 500 mg/kg *F. carica* leaf extract, respectively. Sperm, serum, and bilateral testes tissue samples were examined. Sperm count of the contralateral epididymis and serum follicle-stimulating hormone (FSH) level in the T/D + 250 group were higher than in the T/D and T/D + 125 groups. FSH level in the T/D + 500 group was higher compared to the T/D, T/D + 125, and T/D + 250 groups. Total Oxidant Status (TOS) level in the ipsilateral testis of the T/D + 500 group was lower than that of the T/D, T/D + 125, and T/D + 250 groups. The Oxidative Stress Index (OSI) value in the ipsilateral testis was lower in the T/D + 500 group compared to the T/D, T/D + 125, and T/D + 250 groups. Total Antioxidant Status (TAS) level in the contralateral testis of the T/D + 125, T/D + 250, and T/D + 500 groups was elevated compared to the T/D group. TOS level of the contralateral testis in the T/D + 500 group was lower than that in the T/D and T/D + 125 groups. OSI value of the contralateral testis was lower in the T/D + 125, T/D + 250, and T/D + 500 groups than that in the T/D group. TNF-α and IL-10 immunostaining intensities were improved in bilateral testes of the treatment groups compared to the T/D group. Johnsen’s score of the ipsilateral testis in the T/D + 250 and T/D + 500 groups was greater compared to the T/D group. BAX and CASP3 immunostaining intensities were lower in the bilateral testes of the treatment groups than in the T/D group. BCL-2 immunostaining intensity in bilateral testes of the T/D + 250 and T/D + 500 groups was higher than in the T/D group. By exerting antioxidative, anti-inflammatory, and antiapoptotic effects, *F. carica* leaf extract emerges as a promising therapeutic candidate in testicular ischemia–reperfusion injury. Further studies may help clarify the optimal dosage, potential adverse effects, and fertility outcomes.

## 1. Introduction

Testicular torsion is a urological emergency that primarily affects males under the age of 25, with a higher incidence during neonatal and early adolescent periods [1,2]. Once diagnosed, urgent surgical intervention for detorsion and subsequent orchidopexy of the salvageable testis to ensure reperfusion of the ischemic tissue is required, along with orchidopexy of the contralateral testis [1,2,3,4]. Although detorsion is the only approach for the viability of the torsed testis, it leads to reperfusion injury in addition to ischemic damage elicited by torsion [5,6]. As testicular torsion–detorsion represents a typical ischemia–reperfusion injury, the generation of extensive reactive oxygen species (ROS), the production of proinflammatory cytokines, and the subsequent apoptotic processes are the pathophysiological events underlying the tissue damage and functional loss in cases of testicular torsion–detorsion [5,6,7]. During the torsion period, hypoxia initiates damage through lipid peroxidation, inflammation, and cell death. Subsequently, increased ROS production during the ischemic period intensifies as tissue oxygenation is restored by reperfusion, leading to extensive ROS production that exceeds the tissue’s scavenging capacity [5,7]. ROS production and the ROS-driven increase in proinflammatory cytokines trigger apoptosis in germ cells [5,7,8,9]. Following detorsion, as a result of these damaging processes, testicular atrophy, poor semen quality, and impaired spermatogenesis are observed over time; these disorders are thought to be associated with the duration of ischemia and the oxidative stress that occurs after detorsion [1,2,10]. Poor semen quality and impaired spermatogenesis may not be entirely attributable to unilateral atrophy, as the unaffected contralateral testis is expected to maintain these functions. This condition suggests the possibility of contralateral testicular injury, which has been considered to be associated with the formation of autoantibodies attacking the contralateral testis, reflex sympathetic vasoconstriction leading to impaired perfusion of the contralateral testis, and increased production of molecules related to oxidative stress, ultimately leading to a loss of function [1,8,10,11,12]. Furthermore, experimental and clinical studies have demonstrated histopathological, biochemical, or clinical evidence of contralateral testicular injury after unilateral torsion–detorsion [8,10,12,13,14]. Preservation of the injured testis may result in a smaller contralateral testicular volume, lower sperm count, and decreased motility compared with the corresponding parameters in monorchid patients who underwent orchiectomy after torsion, suggesting alterations in the hormonal axis [14]. Serum luteinizing hormone (LH) and testosterone levels appear not to be affected during long-term follow-up of patients, despite alterations in spermatogenesis, while an increase in serum follicle-stimulating hormone (FSH) levels compared to those in the healthy population is estimated to be related to the deficiency in the inhibitory effects of healthy seminiferous tubules or alterations in the hormonal axis due to the functionally impaired gonad [10,14]. Some experimental and human studies, on the other hand, have reported contrary findings, indicating altered testosterone, FSH, or LH levels following testicular torsion–detorsion [15,16,17,18,19,20,21].

The use of plants as sources of pharmaceutical agents is the focus of numerous experimental studies due to their multiple pharmacological effects, including antioxidant, anti-inflammatory, and metabolic effects. One of these medicinal plants is *Ficus carica* L. (Moraceae), commonly known as the fig, whose bark, leaves, roots, and fruits have been used in various forms for medicinal purposes [22]. Studies have shown that *F. carica* exhibits anti-inflammatory, antioxidant, diuretic, purgative, expectorant, aphrodisiac, anticancer, antimutagenic, antidiabetic, hypolipidemic, reproductive, endocrine, immunological, reno- and hepatoprotective effects [22,23]. In addition to studies demonstrating the antioxidant and hepatoprotective effects of *F. carica* leaf extract [24,25,26,27], other studies have suggested that *F. carica* leaf extract improves experimentally altered testicular structure and function and protects fertility [28,29,30].

The protective effects of *F. carica* leaf extract on sperm parameters and testicular structure have been previously reported in formaldehyde-induced, diabetic, and in vitro spermatogonial stem cell models, while various botanical extracts, such as pomegranate peel extract, have been evaluated in testicular torsion–detorsion models [19,29,30,31,32]. However, to our knowledge, no study has previously combined a long-term bilateral (ipsilateral and contralateral) testicular evaluation with comprehensive phytochemical characterization and a multi-marker immunohistochemical assessment of oxidative, inflammatory, and apoptotic pathways following the administration of *F. carica* leaf extract in a unilateral torsion–detorsion model. To address this gap, we hypothesized that *F. carica* leaf extract would attenuate torsion–detorsion-induced bilateral testicular injury by suppressing oxidative stress, inflammation, and apoptosis, thereby preserving testicular function and germ cell maturation. Therefore, in the current study, we examined the effects of *F. carica* leaf extract on ipsilateral and contralateral testicular tissues with respect to oxidative, inflammatory, and apoptotic processes, as well as on testicular function and germ cell maturation over the long term.

## 2. Results

### 2.1. Results of Phytochemical Analysis

The prepared calibration solutions and the sample were analyzed using an LC–MS Q-TOF system. Data evaluation was performed by examining both positive and negative ionization mode MS data, together with UV detection at 280 nm to monitor the major constituents. Compound identification was carried out based on the obtained mass spectra.

The comparison of the chromatogram obtained from the sample is presented in Figure 1. Quantitative determination was performed only for Bergapten in the sample. Luteolin and Biochanin A were not detected at quantifiable levels. The quantitative results obtained from the calibration curves are provided in Table 1.

Compounds present in the sample and exhibiting peaks in the UV chromatogram were identified based on literature data, the METLIN Metabolite Database, and their MS molecular ions and fragmentation patterns. Detailed information on the identified compounds is provided in Table 2.

### 2.2. Testicular Weight Measurement

The initial and final body weight measurements did not show any significant differences among the groups (*p* = 0.420 and *p* = 0.597, respectively) (Table 3). However, there was a significant difference among the groups in terms of ipsilateral (left) testis weight (*p* < 0.001). Ipsilateral testis weight in the intact group was found to be significantly greater than that in the sham, T/D, T/D + 125, T/D + 250, and T/D + 500 groups (*p* = 0.022, CI: (0.08–0.99); *p* < 0.001, CI: (0.72–1.63); *p* < 0.001, CI: (0.84–1.72); *p* < 0.001, CI: (0.78–1.66); and *p* < 0.001, CI: (0.75–1.66), respectively). Similarly, ipsilateral testis weight in the sham group was significantly higher than that in the T/D, T/D + 125, T/D + 250, and T/D + 500 groups (*p* = 0.010, CI: (0.17–1.10); *p* = 0.002, CI: (0.29–1.19); *p* = 0.005, CI: (0.23–1.13); and *p* = 0.022, CI: (0.19–1.13), respectively) (Table 3).

Statistical analysis also revealed a significant difference among the groups in the mean relative weight of the ipsilateral testis (*p* < 0.001) (Table 3). The relative weight of the ipsilateral testis of the intact group was higher than that in the sham, T/D, T/D + 125, T/D + 250, and T/D + 500 groups (*p* = 0.005, CI: (0.07–0.36); *p* < 0.001, CI: (0.22–0.52); *p* < 0.001, CI: (0.27–0.56); *p* < 0.001, CI: (0.24–0.53); and *p* < 0.001, CI: (0.26–0.55), respectively). Likewise, the mean relative weight of the ipsilateral testis in the sham group was greater than that in the T/D, T/D + 125, T/D + 250, and T/D + 500 groups (*p* = 0.045, CI: (0.01–0.30); *p* = 0.011, CI: (0.04–0.34); *p* = 0.024, CI: (0.02–0.32); and *p* = 0.017, CI: (0.03–0.34), respectively) (Table 3).

There were no statistically significant differences among the groups in terms of contralateral (right) testis weight, as well as the mean relative weight of the contralateral testis (*p* = 0.562 and *p* = 0.804, respectively) (Table 3).

### 2.3. Sperm Count of the Ipsilateral and Contralateral Testes

There was a significant difference among the groups in ipsilateral and contralateral epididymal sperm counts (*p* < 0.001, for both). The ipsilateral epididymal sperm count in the intact group was significantly higher than that in the sham, T/D, T/D + 125, T/D + 250, and T/D + 500 groups (*p* = 0.004, CI: (5.94–28.23); *p* < 0.001, CI: (15.08–37.37); *p* < 0.001, CI: (16.98–38.51); *p* < 0.001, CI: (16.73–38.26); and *p* < 0.001, CI: (19.23–40.77), respectively). Similarly, the ipsilateral epididymal sperm count in the sham group was significantly higher than that in the T/D + 500 group (*p* = 0.024, CI: (1.76–24.06)) (Table 4). The contralateral epididymal sperm count in the intact group was significantly higher than that in the sham group (*p* = 0.026, CI: (0.97–14.73)), while it was significantly lower than that in the T/D + 250 group (*p* = 0.001, CI: (−18.02–−4.72)). On the other hand, the contralateral epididymal sperm count in the sham group was significantly lower than that in the T/D, T/D + 125, T/D + 250, and T/D + 500 groups (*p* = 0.001, CI: (−19.53–−5.32); *p* = 0.001, CI: (−18.48–−4.72); *p* < 0.001, CI: (−26.11–−12.35); and *p* < 0.001, CI: (−19.11–−5.35), respectively). Additionally, the contralateral epididymal sperm count was significantly lower in the T/D and T/D + 125 groups than in the T/D + 250 group (*p* = 0.045, CI: (−13.68–0.07) and *p* = 0.026, CI: (−14.27–−0.97), respectively) (Table 4).

There was also a significant difference among the groups in terms of total sperm count (*p* = 0.002). The total sperm count was significantly greater in the intact group than that in the sham, T/D, T/D + 125, T/D + 250, and T/D + 500 groups (*p* < 0.001, CI: (11.36–38.52); *p* = 0.003, CI: (8.08–35.24); *p* < 0.001, CI: (10.87–37.12); *p* = 0.017, CI: (3.00–29.24); and *p* < 0.001, CI: (12.50–38.74), respectively) (Table 4).

Statistical analysis showed a significant difference among the groups in terms of the presence of ipsilateral epididymal sperm motility (χ2 = 20.874, *p* < 0.001). The presence of sperm motility in the ipsilateral epididymis of rats in the intact group was significantly greater than that in the T/D, T/D + 125, and T/D + 250 groups (χ2 = 5.451, *p* = 0.020; χ2 = 12.173, *p* < 0.001; and χ2 = 9.290, *p* = 0.002, respectively). Similarly, the presence of sperm motility in the ipsilateral epididymis of rats in the sham group was notably greater than in the T/D, T/D + 125, and T/D + 250 groups (χ2 = 4.988, *p* = 0.026; χ2 = 11.193, *p* < 0.001; χ2 = 8.510, *p* = 0.004, respectively) (Table 4). There was no statistically significant difference among the groups in terms of the presence of sperm motility in the contralateral epididymis of rats (Table 4).

### 2.4. Serum Levels of FSH, LH, and Testosterone

There was a significant difference among the groups in terms of FSH levels (*p* < 0.001). Serum FSH levels in the intact group were significantly lower than those in the sham, T/D + 125, T/D + 250, and T/D + 500 groups (*p* = 0.009, CI: (−84.47–−12.48); *p* = 0.009, CI: (−80.51–−11.93); *p* < 0.001, CI: (−124.02–−55.44); and *p* < 0.001, CI: (−170–51–−101.93), respectively). the FSH level was significantly greater in the T/D + 250 group than in the sham, T/D, and T/D + 125 groups (*p* = 0.022, CI: (6.26–77.24); *p* < 0.001, CI: (38.75–109.73); and *p* = 0.014, CI: (9.21–77.79), respectively). Likewise, the FSH level in the T/D + 500 group was significantly higher than that in the sham, T/D, T/D + 125, and T/D + 250 groups (*p* < 0.001, CI: (52.75–123.73); *p* < 0.001, CI: (85.24–156.23); *p* < 0.001, CI: (55.70–124.28); and *p* = 0.009, CI: (12.20–80.78), respectively) (Table 5).

Serum LH and testosterone levels did not show any significant difference among the groups (*p* = 0.079 and *p* = 0.075, respectively) (Table 5).

### 2.5. Serum Levels of TAS, TOS, and PON-1 Activity

Serum TAS and TOS levels, OSI values, and PON-1 activity did not differ significantly among the groups (*p* = 0.080, *p* = 0.240, *p* = 0.253, and *p* = 0.398, respectively) (Table 6).

### 2.6. Bilateral Testicular Tissue TAS and TOS Levels, OSI Values, and PON-1 Activity

According to the assay results, significant differences were observed among the groups in terms of ipsilateral testicular TAS and TOS levels and OSI values (*p* < 0.001, for all) (Table 7).

The TAS level in the ipsilateral testis specimens of the intact group was significantly higher than that in the sham, T/D, T/D + 125, T/D + 250, and T/D + 500 groups (*p* = 0.015, CI: (0.05–0.47); *p* < 0.001, CI: (0.44–0.86); *p* < 0.001, CI: (0.31–0.72); *p* < 0.001, CI: (0.37–0.77); *p* < 0.001, CI: (0.31–0.72), respectively). Similarly, the TAS level in the sham group was significantly higher than that in the T/D, T/D + 125, T/D + 250, and T/D + 500 groups (*p* < 0.001, CI: (0.17–0.61); *p* = 0.015, CI: (0.05–0.46); *p* < 0.001, CI: (0.10–0.52); *p* = 0.017, CI: (0.05–0.46), respectively) (Table 7).

The TOS level in the ipsilateral testis specimens of the T/D + 500 group was significantly lower than that in intact, sham, T/D, T/D + 125, and T/D + 250 groups (*p* < 0.001, CI: (1.62–5.59); *p* < 0.001, CI: (2.28–6.38); *p* < 0.001, CI: (2.67–6.78); *p* < 0.001, CI: (2.10–6.07); and *p* = 0.002, CI: (1.28–5.25), respectively) (Table 7).

The OSI value in the ipsilateral testis specimens of the intact group was significantly lower than that in T/D and T/D + 125 groups (*p* < 0.001, CI: (0.13–0.39); and *p* = 0.010, CI: (0.04–0.30), respectively). Additionally, the OSI value in the T/D group was significantly greater than that in the sham group (*p* = 0.037, CI: (−0.28–0.01)). On the other hand, the OSI value was significantly lower in the T/D + 500 group than that in the sham, T/D, T/D + 125, and T/D + 250 groups (*p* = 0.003, CI: (0.07–0.34); *p* < 0.001, CI: (0.22–0.49); *p* < 0.001, CI: (0.13–0.39); and *p* = 0.001, CI: (0.09–0.35), respectively) (Table 7).

PON-1 activities of the ipsilateral testis specimens were found to be similar among the groups (*p* = 0.161) (Table 7).

There were also statistically significant differences among the groups in terms of contralateral testicular TAS and TOS levels and OSI values (*p* < 0.001, *p* = 0.003, and *p* < 0.001, respectively) (Table 8).

The TAS level in the contralateral testicular specimens of the intact group was significantly higher than that in all torsion–detorsion groups, T/D, T/D + 125, T/D + 250, and T/D + 500 groups (*p* < 0.001, for all). The TAS level in the sham group was significantly higher than that in the T/D, T/D + 125, T/D + 250, and T/D + 500 groups (*p* < 0.001, CI (0.55–0.90); *p* < 0.001, CI: (0.36–0.70); *p* < 0.001, CI: (0.31–0.65); and *p* = 0.037, CI: (0.17–0.51), respectively). On the other hand, the TAS level in the T/D group was notably lower compared to T/D + 125, T/D + 250, and T/D + 500 groups (*p* = 0.034, CI: (−0.37–−0.01); *p* < 0.001, CI: (−0.42–−0.06); and *p* = 0.009, CI: (−0.56–−0.20), respectively). Additionally, the TAS level in the contralateral testicular specimens of the T/D + 125 group was lower than that in the T/D + 500 group (*p* = 0.031, CI: (0.01–0.36)) (Table 8).

The TOS level in the contralateral testicular samples of the T/D group was significantly greater than that in the intact group (*p* = 0.002). The TOS level in the T/D + 500 group was significantly lower than that in the sham, T/D, and T/D + 125 groups (*p* = 0.010, CI: (0.47–3.37); *p* < 0.001, CI: (1.55–4.46); and *p* = 0.012, CI: (0.42–3.22), respectively) (Table 8).

The OSI value in the contralateral testicular samples of the intact group was significantly lower than that in the T/D, T/D + 125, and T/D + 250 groups (*p* < 0.001, CI: (0.22–0.44); *p* < 0.001, CI: (0.09–0.30); and *p* = 0.019, CI: (0.02–0.23), respectively). The OSI value in the T/D group was significantly higher than that in the sham group (*p* < 0.001, CI: (−0.35–−0.13)). The OSI value was significantly lower in the T/D + 125, T/D + 250, and T/D + 500 groups than that in the T/D group (*p* = 0.016, CI: (0.02–0.24); *p* < 0.001, CI: (0.10–0.31); and *p* < 0.001, CI: (0.20–0.41), respectively). Additionally, the OSI value in the T/D + 500 group was significantly lower than that in the T/D + 125 group (*p* = 0.002, CI: (0.06–0.27)) (Table 8).

There was no statistically significant difference among the groups in terms of PON-1 activity in the contralateral testicular specimens (*p* = 0.740) (Table 8).

### 2.7. Histological Findings in Bilateral Testicular Tissue

Evaluation of Johnsen’s scores in both the ipsilateral and contralateral testicular specimens revealed significant differences among the groups (*p* < 0.001 for both) (Table 9).

Johnsen’s score in the ipsilateral testicular tissue in the intact group was significantly greater than that in the T/D, T/D + 125, T/D + 250, and T/D + 500 groups (*p* < 0.001, CI: (2.34–4.54); *p* < 0.001, CI: (1.90–4.03); *p* < 0.001, CI: (0.97–3.09); and *p* = 0.001, CI: (0.77–2.89), respectively). Similarly, Johnsen’s score in the ipsilateral testicular specimens of the sham group was significantly higher than that in the T/D, T/D + 125, and the T/D + 250 groups (*p* < 0.001, CI: (1.58–3.85); *p* < 0.001, CI: (1.14–3.36); and *p* = 0.021, CI: (0.21–2.41), respectively). However, Johnsen’s score of the ipsilateral specimens in the T/D group was notably lower compared to the T/D + 250 and T/D + 500 groups (*p* = 0.013, CI: (−2.50–−0.30); and *p* = 0.005, CI: (−2.70–−0.51), respectively). Additionally, Johnsen’s score in the ipsilateral testis of the T/D + 125 group was significantly lower than that in the T/D + 500 group (*p* = 0.037, CI: (−2.19- 0.07)) (Table 9) (Figure 2a).

Johnsen’s score in the contralateral testis of the intact group was significantly higher than that in all other groups (*p* < 0.001 for all) (Table 9) (Figure 2b).

Consistent with Johnsen’s score findings, the ipsilateral testicular tissue of the intact group exhibited uniform, round-to-oval-shaped, seminiferous tubules, with a regular multilayered germinal epithelium and lumina containing many spermatozoa. In the T/D group, ipsilateral testicular tissue samples showed prominent tubular disorganization, disrupted uniform shape through the tubular profiles, and atrophy with notable attenuation and disorganization of germinal epithelium. In some tubules, the germinal epithelium appeared to be completely distorted, and some tubular lumina contained only sloughed cellular aggregates rather than mature germ cells. In the ipsilateral testicular specimens of the treatment groups, particularly in 250 and 500 mg/kg groups, germinal epithelial thickness and organization were partially restored, compared to the T/D group, and well-organized spermatogenic cell layers as well as spermatozoa-containing lumina are visible in a greater number of seminiferous tubules, although the histoarchitecture did not fully restore to the appearance observed in the intact group (Figure 2a).

Regarding the contralateral testicular tissue, a slight tubular atrophy with a slight attenuation in germinal epithelium and in dimensions of the seminiferous tubules was observed, affecting a greater number of tubules in the T/D group, rather than a smaller number of tubules in the treatment groups, along with normal appearance of tubular profiles in the intact and sham groups (Figure 2b).

### 2.8. Immunohistochemical Findings in Bilateral Testicular Tissue

There was a significant difference among the groups in terms of TNF-α immunostaining intensity in the ipsilateral testicular tissue specimens (*p* = 0.012). TNF-α immunostaining intensity in the ipsilateral testicular specimens of the T/D group was significantly higher than that in the intact and sham groups (*p* < 0.001 for both, CI: (−21.55–−7.30; −16.20–−1.49), respectively). Additionally, TNF-α immunostaining intensity in the ipsilateral testis samples of the T/D + 125, T/D + 250, and T/D + 500 groups was significantly lower than that in the T/D group (*p* < 0.001 for all, CI: (1.64–15.89; 1.88–16.36; 1.39–15.64), respectively) (Table 10) (Figure 3a,b).

IL-10 immunostaining intensity in the ipsilateral testis samples was also significantly different among the groups (*p* = 0.009). IL-10 immunostaining intensity in the ipsilateral testis of the T/D group was significantly lower than that in the intact group (*p* = 0.012, CI: (−0.29–17.55)). IL-10 immunostaining intensity in the ipsilateral testis was significantly higher in the T/D + 250 and T/D + 500 groups than that in the sham group (*p* < 0.001 for both, CI: (0.56–18.39; 2.66–20.50), respectively). Additionally, IL-10 immunostaining intensity in the ipsilateral testis was significantly higher in the T/D + 125, T/D + 250, and T/D + 500 groups than in the T/D group (*p* < 0.001, all, CI: (0.87–18.71; 5.08–22.92; 7.18–25.02), respectively) (Table 10) (Figure 3a,b).

Comparison of BAX immunostaining intensity in the ipsilateral testis revealed a significant difference among the groups (*p* < 0.001). BAX immunostaining intensity in the ipsilateral testis was significantly higher in the sham, T/D, T/D + 125, and T/D + 250 than in the intact group (*p* < 0.001 for all, CI: (−8.63–−1.28; −13.69–−6.34; −8.65–−1.56; −7.83–−0.74; −4.39–2.70), respectively). Similarly, BAX immunostaining intensity in the ipsilateral testis specimens of the T/D group was significantly higher than that in the sham group (*p* < 0.001, CI: (−8.85–−1.26)). BAX immunostaining intensity in the ipsilateral testis of the T/D + 125, T/D + 250, and T/D + 500 groups was significantly lower than that in the T/D group (*p* < 0.001, all, CI: (1.23–8.58; 2.05–9.40; 5.50–12.85), respectively). Additionally, BAX immunostaining intensity in the ipsilateral testis of the T/D + 500 group was f significantly lower than that in the sham group (*p* < 0.001, CI: (0.44–7.79)) (Table 10) (Figure 3a,b).

BCL-2 immunostaining intensity in the ipsilateral testis specimens was significantly different among the groups (*p* = 0.035). BCL-2 immunostaining intensity in the ipsilateral testis specimens of the T/D group was significantly lower than that in the intact and sham groups (*p* = 0.012, CI: (5.19–26.37); and *p* = 0.009, CI: (7.21–28.25), respectively). In contrast, BCL-2 immunostaining intensity in the ipsilateral testis of the T/D + 250 and T/D + 500 groups was significantly higher than that in the T/D group (*p* < 0.001 for both, CI: (−25.93–−4.74; −25.18–−4.00), respectively) (Table 10) (Figure 3a,b).

There was a significant difference among the groups in terms of CASP3 immunostaining intensity in the ipsilateral testis specimens (*p* < 0.001). CASP3 immunostaining intensity in the ipsilateral testis specimens of the sham, T/D, and T/D + 125 groups was significantly higher than that in the intact group (*p* < 0.001 for all, CI: (−11.80–−2.65; −18.18–−9.03; −9.98–−1.14), respectively). CASP3 immunostaining intensity in the ipsilateral testis specimens was also significantly higher in the T/D group than that in the sham group (*p* < 0.001, CI: (−11.14–−1.65)). In contrast, CASP3 immunostaining intensity in the ipsilateral testis of the T/D + 125, T/D + 250, and T/D + 500 groups was significantly lower than that in the T/D group (*p* < 0.001 for all, CI: (5.21–14.36; 8.53–17.68; 3.47–12.62), respectively). Additionally, CASP3 immunostaining intensity in the ipsilateral testis of the T/D + 500 group was significantly lower than that in the sham and T/D + 125 groups (*p* = 0.005, CI: (2.15–11.30); and *p* < 0.001, CI: (0.64–9.48), respectively) (Table 10) (Figure 3a,b).

There was a statistically significant difference among the groups in terms of TNF-α immunostaining intensity in the contralateral testicular tissue specimens (*p* < 0.001). TNF-α immunostaining intensity in the contralateral testis was significantly higher in the T/D group than that in the intact and sham groups (*p* < 0.001 for both, CI: (−10.01–−4.93; −8.23–−2.98), respectively). On the other hand, the TNF-α immunostaining intensity in the contralateral testis was significantly lower in the T/D + 125, T/D + 250, and T/D + 500 groups than that in the T/D group (*p* < 0.001 for all, CI: (2.92–8.00; 3.84–8.92; 5.89–10.97), respectively). Additionally, TNF-α immunostaining intensity in the contralateral testis in the T/D + 500 group was significantly lower than that in the sham and T/D + 125 groups (*p* = 0.030, CI: (0.28–5.36); and *p* = 0.019, CI: (0.51–5.41), respectively) (Table 11) (Figure 4a,b).

IL-10 immunostaining intensity in the contralateral testicular tissue differed significantly among the groups (*p* = 0.002). IL-10 immunostaining intensity in the contralateral testicular tissue in the sham and T/D groups was significantly lower than that in the intact group (*p* = 0.004, CI: (−20.43–4.26); and *p* < 0.001, CI: (−25.83–−9.67), respectively). However, IL-10 immunostaining intensity in the contralateral testis of the T/D + 125, T/D + 250, and T/D + 500 groups was significantly higher than that in the T/D group (*p* = 0.007, CI: (3.32–7.33); *p* = 0.005, CI: (3.79–19.95); and *p* = 0.002, CI: (4.91–21.07), respectively) (Table 11) (Figure 4a,b).

BAX immunostaining intensity in the contralateral testicular tissue specimens of the groups differed significantly among the groups (*p* < 0.001). BAX immunostaining intensity in the contralateral testis was significantly higher in the T/D and T/D + 125 groups than that in the intact group (*p* < 0.001, CI: (−18.41–−9.04); and *p* = 0.036, CI: (−9.37–−0.32), respectively). BAX immunostaining intensity in the contralateral testis of the T/D group was significantly higher than that in the sham group (*p* < 0.001, CI: (−16.03–−6.36)). BAX immunostaining intensity in the contralateral testis was significantly lower in the T/D + 125, T/D + 250, and T/D + 500 groups than that in the T/D group (*p* < 0.001, for all, CI: (4.19–13.56; 5.20–14.57; 8.66–18.04), respectively) (Table 11) (Figure 4a,b).

There was a statistically significant difference among the groups in terms of BCL-2 immunostaining intensity in the contralateral testicular tissue specimens (*p* = 0.018). BCL-2 immunostaining intensity in the contralateral testis was significantly lower in the T/D and T/D + 125 groups than that in the intact group (*p* < 0.001, CI: (−16.77–−4.84); and *p* = 0.040, CI: (−11.83––−0.30), respectively). BCL-2 immunostaining intensity in the contralateral testis was significantly higher in the T/D + 250 and T/D + 500 groups than that in the T/D group (*p* = 0.012, CI: (1.83–13.76); and *p* = 0.014, CI: (1.63–13.56), respectively) (Table 11) (Figure 4a,b).

CASP3 immunostaining intensity in the contralateral testicular tissue differed significantly among the groups (*p* < 0.001). CASP3 immunostaining intensity in the contralateral testis of the T/D group was significantly higher than that in the intact and sham groups (*p* < 0.001, CI: (−14.46–−6.31); and *p* = 0.004, CI: (−10.58–−2.16), respectively). CASP3 immunostaining intensity in the contralateral testis specimens was significantly lower in the T/D + 125, T/D + 250, and T/D + 500 groups than that in the T/D group (*p* < 0.001 for all, CI: (3.82–11.96; 4.02–12.16; 4.63–12.78), respectively) (Table 11) (Figure 4a,b).

## 3. Discussion

Testicular torsion interrupts blood circulation, which may cause loss of the gonad unless immediate intervention is performed to reestablish blood flow to the organ, and detorsion surgery is performed to restore blood flow if the torsed testicular tissue appears salvageable [4,15,33]. Although detorsion is essential for preserving the viability of the torsed testis, it may lead to reperfusion injury in the ischemic tissue, causing testicular oxidative stress, germ cell apoptosis, and impaired spermatogenesis [1,18,33]. To develop potential therapeutic approaches to prevent such deterioration, numerous experimental studies have investigated effective strategies targeting the pathophysiological mechanisms underlying testicular ischemia–reperfusion injury [31,34].

A previous study by Onishi and colleagues on the protective effects of dimethyl fumarate against torsion–detorsion injury demonstrated significantly elevated MDA levels and reduced SOD and GSH levels in testicular samples after 1.5 h of torsion and 7 days of detorsion [35]. Likewise, Shokoohi and colleagues investigated the effects of *Fumaria parviflora* on testicular injury and found that SOD and glutathione peroxidase (GPx) levels were significantly reduced while MDA levels were significantly elevated in the serum samples of rats 14 days after detorsion following a 4 h unilateral testicular torsion [21]. Furthermore, in a study by Cvetkovic et al., the oxidant and antioxidant status of serum and bilateral testicular tissue was investigated in rats subjected to a 1 h unilateral testicular torsion followed by a 30-day detorsion period. Elevated concentrations of advanced oxidation protein products (AOPP), increased levels of thiobarbituric acid reactive substances (TBARS), and decreased concentrations of total sulfhydryl (T-SH) groups were detected in the plasma, suggesting a systemic effect of unilateral testicular torsion–detorsion, despite the prolonged reperfusion period [36]. This suggestion was also supported by the higher catalase (CAT) activity, TBARS concentrations, and GSH levels in the ipsilateral testis, and higher TBARS concentrations in the contralateral testis, supporting the systemic effects of unilateral torsion on the contralateral testis [36]. Additionally, the researchers concluded that reperfusion significantly induced antioxidative defenses, leading to increased CAT activity and GSH levels in the ipsilateral testis despite the increased TBARS concentrations [36]. More importantly, in a study by Oral et al. examining the effects of oral zinc administration on long-term injury to the ipsilateral and contralateral testes, elevated MDA levels and decreased GSH and SOD levels were reported in both ipsilateral and contralateral testicular tissues during the first, second, and even third months after detorsion, indicating prolonged oxidative stress [37]. The effect of *F. carica* leaf extract in alleviating reperfusion-induced arrhythmias and reducing infarct size in an ex vivo cardiac ischemia–reperfusion study was suggested to be related to its antioxidant capacity [26]. Additionally, a previous study showed that *F. carica* leaf extract administration mitigated histopathological and biochemical alterations, thereby preventing hepatic injury and improving oxidative stress parameters, including glutathione and MDA levels, as well as SOD and catalase activities, in animal models of carbon tetrachloride-induced hepatotoxicity [27]. Similarly, in a study exploring the effects of *F. carica* leaf extract in a cisplatin-induced nephrotoxicity model, it was demonstrated that oral administration of *F. carica* leaf extract reduced tissue MDA levels and increased tissue glutathione (GSH), nitric oxide (NO), and total antioxidant capacity (TAC) levels, which had been impaired by cisplatin-induced ROS generation [38]. Additionally, in a study designed to investigate the effects of the extracts from different parts of the *F. carica* on type-1 diabetes and its complications, it was demonstrated that *F. carica* leaf extract increased GSH levels and CAT and GPx activities, while decreasing MDA levels in liver, kidney, and pancreatic tissue samples of diabetic rats [39]. In contrast to the previous studies demonstrating an improvement in the serum oxidant–antioxidant status, serum TAS, TOS, OSI, and PON values did not show statistically significant changes, although serum TOS levels and OSI values tended to improve in *F. carica* leaf extract-administered groups. However, the results of the bilateral testicular tissue revealed significant improvements in oxidant–antioxidant status localized to testicular tissue. Ipsilateral testicular TOS levels and OSI values revealed a significant improvement in testicular oxidative status following administration of 500 mg/kg *F. carica* leaf extract. Similarly, contralateral testicular evaluation showed dose-dependent improvements in TAS, TOS, and OSI values in the treatment groups, although TOS values did not reach statistical significance in the 125 mg/kg and 250 mg/kg *F. carica* leaf extract treatment groups. It is also noteworthy that while contralateral testicular TAS, TOS, and OSI values improved progressively across the three doses, the improvement in ipsilateral testicular TAS values followed a trend reaching greater values at the 125 mg/kg and 500 mg/kg doses than the 250 mg/kg dose, rather than following a gradual, dose-proportional trend, even though there was not a statistically significant difference. Additionally, although the oxidative stress panel used in this study (TAS, TOS, OSI, and PON-1) provides a direct, composite assessment of overall redox balance rather than a specific characterization of oxidative damage or antioxidant defense mechanisms, future studies on effects of *F. carica* leaf extratc in testicular torsion–detorsion models incorporate further assessement of biomarkers, such as MDA, GSH, and the activities of SOD, CAT, and GPx are warranted to provide a more comprehensive characterization of the underlying oxidative damage and antioxidant defense mechanisms.

In a study by Ertürk et al., focusing on the impact of bone marrow-derived mesenchymal stem cells in rats with testicular torsion, torsion–detorsion resulted in significantly lower IL-10 levels in the ipsilateral testis 7 days after detorsion, while TNF-α levels increased, although the difference was not statistically significant [40]. Furthermore, Onishi and colleagues reported drastically elevated mRNA expression of nuclear factor kappa B (NF-κB), TNF-α, IL-1β, and IL-6 in testicular tissue exposed to a 1.5 h torsion followed by a 7-day detorsion period [35]. *F. carica* leaves contain several compounds, including bergapten and lupeol, with regulatory effects on inflammation [41]. An experimental study investigating the anti-inflammatory effects of *F. carica* leaf extract demonstrated that inflammatory cell infiltration, exudate volume, granulation tissue weight, and inflammation-related mediators, including TNF-α and prostaglandin E2 (PGE2), were all alleviated at the inflammation site following *F. carica* leaf extract administration. And these anti-inflammatory effects were suggested to be relevant to its free radical-scavenging activity [42]. Furthermore, Dai et al. demonstrated that *F. carica* extract treatment in mice subjected to unilateral sciatic nerve denervation decreased the expression levels of the proinflammatory cytokines IL-1β and IL-6 in atrophic skeletal muscle. This anti-inflammatory property of the extract was shown to depend on its effects on the activation of peroxisome proliferator-activated receptor alpha (PPARα) and the inhibition of NF-κB [43]. In a study examining the anti-inflammatory effects of *F. carica* leaf extract on wound healing, *F. carica* leaf extract significantly decreased the expression of the inflammatory markers TNF-α, IL-1β, and IL-6 in the skin area around the wound [44]. We evaluated the anti-inflammatory effect of *F. carica* leaf extract by TNF-α and IL-10 immunoreactivity in the testicular tissue sections. Our results, which showed significant improvements in the immuno-expression of TNF-α and IL-10 in the ipsilateral testicular specimens, were consistent with these previous studies. Furthermore, analyses of contralateral testicular specimens revealed significant attenuation of TNF-α immuno-expression and a significant elevation in IL-10 immuno-expression, which may indicate that the anti-inflammatory effect of the extract is not confined to the directly injured tissue but extends systemically to the contralateral gonad. Although these results are consistent with previous reports, more extensive studies detailing the interaction of *F. carica* leaf extract with the molecular machinery of inflammation, such as NF-κB, should be designed.

Torsion and subsequent detorsion lead to atrophy in the ipsilateral testis over time [10,14,16,17,45]. The main functions, the production of germ cells and the release of testosterone, are affected by this condition in the long term, as demonstrated by numerous studies [1,10,18,19,45]. In addition to germ cell injury and eventual atrophy in the twisted testis, impaired germ cell production may also occur in the contralateral testis, as evidenced by human and experimental studies [16,46]. Cosentino and colleagues investigated the histological outcomes of various durations of unilateral testicular torsion in the contralateral testis and found that even an hour of unilateral testicular torsion led to a significant loss of sperm and spermatids in the seminiferous tubules of the contralateral testis [46]. In a study evaluating the effects of pomegranate peel extract on testicular torsion–detorsion injury, it was demonstrated that seminiferous tubules of the testicular tissue of the rats subjected to a 90 min period of torsion followed by 60 days of reperfusion exhibited drastic atrophy with loss of germ cells and Sertoli cells in some tubules, resulting in a very low Johnsen’s score [19]. In another study exploring the effects of royal jelly on testicular torsion-induced ischemia–reperfusion injury in rats, it was found that the testes of rats subjected to 90 min of torsion exhibited significantly lower Johnsen’s score after a 50-day reperfusion period, indicating reduced spermatogenesis index [20]. Similarly, in the study by Shokoohi et al., a 4 h unilateral testicular torsion followed by a 14-day reperfusion period led to a significant reduction in spermatogenesis, as evidenced by a low Johnsen’s score. Furthermore, this finding was supported by increased BAX and decreased BCL-2 gene expression, and by a higher number of TUNEL-positive cells in the affected testis, suggesting oxidative stress that triggers apoptosis in germ cells [21]. Compounds from *F. carica* leaf extract also modulate apoptotic processes. As noted by Zhang et al., administration of *F. carica* leaf extract to diabetic mice resulted in increased levels of the antiapoptotic protein BCL-2 and decreased expression of the apoptotic proteins BAX, cytochrome c, cleaved caspase 3, and cleaved caspase 8 in pancreatic tissue [47]. Additionally, a study by Hassan et al. revealed that orally administered *F. carica* leaf extract reduced MDA levels while improving GSH levels and SOD activity in liver and kidney tissue samples of rats exposed to monosodium glutamate or metanil yellow. Moreover, this study demonstrated the antiapoptotic effects of the extract by reducing BAX and caspase-3 expression and increasing BCL-2 expression in liver and kidney specimens from rats with injury induced by monosodium glutamate or metanil yellow [48]. Consistent with these studies, *F. carica* leaf extract, administered at doses of 250 mg/kg and 500 mg/kg, significantly increased the Johnsen’s score of the ipsilateral testis. Further immunohistochemical analysis of BAX, BCL-2, and CASP3 expression in bilateral testicular specimens demonstrated that 250 mg/kg and 500 mg/kg *F. carica* leaf extract administration significantly increased BCL-2 expression in both ipsilateral and contralateral testes, while all three doses significantly decreased BAX and CASP3 expression in bilateral testicular tissues. Beyond the studies mentioned above, which evaluated Johnsen’s score or apoptotic markers predominantly in the ipsilateral or directly injured tissue [19,20,21], the present findings extend the current understanding of *F. carica* leaf extract’s antiapoptotic activity by demonstrating a parallel effect in the contralateral testis, supporting a bilateral, rather than strictly local, mode of protection.

The spermatogenic function of the testis is shown to be affected by testicular torsion in the chronic period [10,15,16,19,45]. In cases of impaired germ cell production, sperm counts and motility were found to be decreased [1,10,15,45,49]. In the study by Beigi Boroujeni et al., the sperm count and the percentage of viable sperm were found to be apparently diminished 60 days after detorsion in the epididymis of rats, which may result from the destruction and apoptosis of germ cells lining the seminiferous tubules [19]. As reported by Shokoohi and colleagues, torsion and detorsion of the testis resulted in reduced sperm concentration and decreased motility [21]. Beyond its various documented benefits, the beneficial effects of *F. carica* leaf extract on altered testicular function have also been demonstrated in studies using different animal models. In one of these studies, Naghdi et al. investigated the effects of *F. carica* leaf extract on testicular morphology and sperm parameters in an experimental model of formaldehyde intoxication. They found that *F. carica* leaf extract improved sperm count and nonprogressive motility and ameliorated the spermatogenic arrest in the seminiferous tubules, all of which were adversely affected by formaldehyde intoxication [30]. In another study exploring the beneficial effects of *F. carica* leaf extract on sperm parameters in diabetic rats, Abu Bakar et al. reported that, in addition to its antihyperglycemic effects, which prevent diabetes-related complications that alter sperm quality parameters, *F. carica* leaf extract induced the expression of unique proteins associated with fertility-related functions [29]. Similarly, Fahmy et al. demonstrated that administration of *F. carica* leaf extract in a mouse model of cisplatin-induced genotoxicity reduced testicular injury in a dose-dependent manner and restored spermatogenesis [28]. The fertility-protective and fertility-improving effects of *F. carica* leaf extract were attributed to its bioactive compounds, which possess antioxidant properties, as well as to its saponin content, which exhibits actions similar to those of testosterone [29,30]. In the current study, sperm counts on the torsed side epididymis of the animals from the torsion–detorsion and treatment groups did not show a significant difference; however, sperm counts on the contralateral side improved significantly in the 250 mg/kg treatment group, and total sperm count appeared to be increased, although the difference did not reach statistical significance, compared to untreated animals. Notably, this improvement in contralateral epididymal sperm count did not increase further at the 500 mg/kg dose, indicating a non-monotonic, rather than strictly dose-dependent response for this parameter, as also seen in the total sperm count. Regarding sperm motility in the ipsilateral epididymis, the 500 mg/kg treatment group appeared to approach the intact and sham animals, as no statistically significant difference was observed among these groups. The present study assessed only sperm count and the presence/absence of motility, while sperm morphology, viability, germ cell DNA integrity, and functional fertility outcomes verified by mating trials, pregnancy rates, and litter size were not evaluated. The improvements observed in oxidative stress, histological, and immunohistochemical parameters indicate attenuation of injury and the protection of testicular tissue, but do not necessarily demonstrate the restoration of functionality and fertility. Therefore, comprehensive spermatozoal parameters, genomic integrity of germ cells, and dedicated fertility assays, which were not addressed in the current study, could be further evaluated in future studies to determine whether *F. carica* leaf extract has additional effects on the genetic and morphological features of spermatozoa.

Research investigating the effects of testicular torsion in humans has focused on FSH, LH, and testosterone levels as well as semen analysis and testicular biopsies [10,15,16,45]. A contradictory situation exists between the endocrine and germ cell production functions of testicular tissue in patients who have undergone testicular torsion [1,10,15]. Accordingly, some studies have reported normal testosterone levels, indicative of preserved endocrine function, and normal LH levels, consistent with its regulatory role in testicular endocrine function, whereas elevated FSH levels have been associated with impaired spermatogenesis [10,14]. In contrast, some experimental and human studies have reported elevated FSH and LH levels, as well as altered testosterone levels [15,16,17,18]. In an experimental study by Turner et al., testicular venous plasma testosterone levels 30 days after detorsion were found to be greatly reduced in rats subjected to 1 h unilateral torsion compared with control rats [18]. Furthermore, in the study by Beigi Boroujeni et al., significantly reduced testosterone levels in the blood samples of animals after a 90 min testicular torsion followed by a 60-day reperfusion were shown [19]. Similarly, a significant reduction in blood testosterone levels was observed in a rat model subjected to 90 min of torsion and a 50-day reperfusion period [20]. Likewise, serum levels of FSH, LH, and testosterone were evaluated in the present study. There was no statistically significant difference among the groups in LH and testosterone levels, despite slightly increased levels in the 250 mg/kg treatment group. Notably, these hormone levels demonstrated a decrease rather than sustained increase at the 500 mg/kg dose. However, we found a significant increase in FSH levels in the 250 mg/kg and 500 mg/kg *F. carica* leaf extract treatment groups. This dose-dependent elevation in FSH was accompanied by improvements in oxidative stress markers and Johnsen’s scores, and reduced apoptotic markers in the same treatment groups, without concurrent changes in LH or testosterone. Physiologically, FSH secretion is regulated largely by Sertoli-cell-derived inhibin B, whereas LH and testosterone are governed by a distinct, largely independent feedback loop acting predominantly through Leydig cell steroidogenesis [50,51]. Therefore, an isolated rise in FSH may reflect a compensatory pituitary response to reduced inhibin B secretion secondary to impaired Sertoli-cell or seminiferous tubular function [10,14]. However, it was reported that different cell populations of the testicular tissue (germ cells, Sertoli cells, and Leydig cells) possess distinctive vulnerabilities to different durations of torsion, with germ cells being most vulnerable; and 1 h 720° torsion is adequate to generate spermatogenic deterioration, germ cell injury and apoptosis in rat models, while Leydig cells are more resistant, providing a considerable steroid synthesis capacity [34,52]. On the other hand, the saponin content of *F. carica* leaf extracts has been speculated to exert testosterone-like actions and good effects on FSH levels [29,32,53]. Such a mechanism, if present, could act directly on Sertoli or germ cell populations independently of the classical LH–testosterone loop. These suggestions regarding the differential vulnerability of testicular cell populations to ischemia and also the saponin content of *F. carica* leaf extracts are more likely to support our finding that Johnsen’s score of the ipsilateral testis revealed significant improvement in the 250 and 500 mg/kg treatment groups; also, the sperm count of the contralateral testis in the 250 mg/kg treatment group showed a significant elevation, increasing the total sperm count, although it did not reach significant levels. Nonetheless, because inhibin B levels and other mechanistic hormonal assays, as well as the saponin content of the extract, were not evaluated in the present study, both suggestions remain to be explored in further investigations, and the physiological significance of the observed FSH elevation should be clarified in future studies, incorporating a more comprehensive evaluation of the hypothalamic–pituitary–gonadal axis.

The primary pathophysiological driver of testicular torsion is ischemia–reperfusion (I/R) injury, and the onset of the ischemic phase triggers lipid peroxidation and hypoxia, while subsequent detorsion paradoxically exacerbates tissue damage through excessive production of proinflammatory cytokines and ROS. These molecular events culminate in germ cell apoptosis, testicular atrophy, and impaired spermatogenesis, and may also affect the contralateral testis through reflex sympathetic vasoconstriction—thereby impairing perfusion of the contralateral testis—as well as systemic oxidative stress and autoimmune responses against sperm antigens [1,8,10,11,12]. Drawing our attention to this perspective, we additionally investigated the effects of *F. carica* leaf extract on the contralateral testicular tissue in the present study. Although we did not investigate the precise mechanism underlying contralateral testicular injury following unilateral torsion–detorsion, such as evaluating contralateral testicular blood flow during experimental procedures or investigating the presence of autoantibodies in the present study, we observed significant alterations in the TAS, TOS, OSI values, Johnsen’s scores, and TNF-α, IL-10, BAX, BCL-2, and CASP3 immunoreactivities of contralateral testicular specimens of the animals from torsion–detorsion group. These findings may be interpreted as contralateral effects of unilateral torsion–detorsion; however, the mechanistic explanations discussed above should be regarded as hypothetical within the context of the present study, and dedicated experimental approaches (e.g., assessment of circulating autoantibodies, systemic inflammatory mediators, or contralateral blood flow) are needed to clarify the underlying pathophysiology of contralateral injury.

The use of medicinal plants as adjunctive therapies has gained significant attention due to their multi-targeted antioxidant and anti-inflammatory properties and their effects on pathways involved in ischemia–reperfusion injury. The literature suggests that *F. carica* leaf extract exerts its protective effects through several key mechanisms. In vitro evidence indicates that *F. carica* leaf extract supports the stemness and proliferation of spermatogonial stem cells in a dose-dependent manner without inducing cytotoxicity. This regenerative support is vital for restoring the germ cell population post-I/R injury [32]. *F. carica* has been shown to alleviate overexpression of inflammatory markers, including iNOS and NF-κB, while reducing the immunoreactivity of cytokines such as TNF-α. *F. carica* extract likely mitigates the intrinsic apoptotic cascade triggered during the reperfusion phase by modulating the BAX/BCL-2 ratio [28]. Previous in vivo studies have demonstrated that *F. carica* leaf extract significantly improves sperm count, motility, and the gonadosomatic index in tissues subjected to chemical or oxidative stress, with spermatogenic arrest rarely observed after treatment [30]. Treatment with *F.carica* leaf extract did not fully restore all parameters, including the absence of significant improvement in ipsilateral testicular weight, ipsilateral and total sperm counts, contralateral Johnsen’s score, or serum oxidative markers and tissue PON-1 activity. On the other hand, findings relevant to oxidative status, inflammatory and apototic states of ipsilateral and contralateral testicular tissues, as well as ipsilateral Johnsen’s score in the present study are consistent with the reported antioxidative, anti-inflammatory, and antiapoptotic effects of *F. carica* leaf extract, although these selective effects—rather than global, affecting specific oxidative, inflammatory, and apoptotic parameters without fully normalizing testicular structure, sperm output, or systemic oxidative status—require further confirmation through mechanistic studies examining the effects of extract on NF-κB signaling, Nrf2 activation, MAPK pathways, or mitochondrial apoptotic signaling by measuring the protein levels and gene expression levels relevant to these pathways.

A significant finding of the phytochemical analysis was the presence of bergapten, a furanocoumarin previously identified among the characteristic constituents of *F. carica* leaves [54,55]. Bergapten has been associated with antioxidant and cytoprotective effects, including the attenuation of oxidative stress and preservation of mitochondrial function. These properties may be relevant to testicular torsion–detorsion injury, which involves excessive ROS generation, lipid peroxidation, and mitochondrial dysfunction [56]. However, the present findings do not allow the protective effects of the extract to be attributed specifically to bergapten. Rather, bergapten may represent one of several constituents contributing to the observed biological activity. This interpretation is particularly important because luteolin and biochanin A were detected below the limit of quantification, while several other compounds were only tentatively identified. Therefore, the contribution of individual constituents, as well as possible additive or synergistic interactions among them, remains unclear. Previous studies have demonstrated considerable variation in the furanocoumarin composition of *F. carica* leaves among different varieties, supporting the need for further phytochemical characterization [55].

On the other hand, batch-to-batch standardization was not performed in the present study. Although the extract used in the experiments was prepared from the same batch of authenticated plant material under the same extraction conditions, this does not establish reproducibility across independent batches. Batch-to-batch variability is a recognized challenge in botanical extracts and may affect both chemical composition and biological activity [57,58]. Accordingly, future studies should incorporate chromatographic fingerprinting and quantitative marker-based standardization across independent batches to improve reproducibility. Furthermore, bioactivity-guided fractionation and the evaluation of individual constituents and their combinations will be required to determine which compounds contribute to the protective effects observed in the present study.

Several additional limitations of the experimental design should be acknowledged. An extract-only group (*F. carica* leaf extract-administered without torsion–detorsion) was not included in the present design, in accordance with efforts to minimize animal numbers. The inclusion of an extract-only group in future studies would enable more direct assessment of the extract’s intrinsic safety profile. Additionally, this study evaluated a single terminal time point (56 days after detorsion), which does not allow distinction between acute cytoprotective effects and longer-term chronic tissue remodeling; future studies incorporating multiple time points would help clarify the temporal dynamics of the observed protective effects of *F. carica* leaf extract.

## 4. Materials and Methods

### 4.1. Plant Material and Extraction

Leaves of *F. carica* were collected from Seferihisar in July 2020, and a voucher specimen was deposited in the Herbarium of Gazi University Faculty of Pharmacy (GUE 3852). The air-dried leaves were powdered, and 511.32 g of the material was extracted with 6 L of 80% methanol at room temperature. Extraction was performed six times at two-day intervals, with each cycle involving 30 min of stirring using a magnetic stirrer. The combined aqueous–methanolic extracts were concentrated to dryness under reduced pressure at 40 °C using a rotary evaporator (yield: 31.49%).

### 4.2. Phytochemical Analysis

The analyses were carried out using an Agilent 1260 Infinity HPLC System coupled to an Agilent 6550 iFunnel Q-TOF Mass Spectrometer (Agilent Technologies, Inc., Santa Clara, CA, USA). The MS system was operated using dual-spray electrospray ionization with an Agilent Jet Stream source, and analyses were performed in both positive and negative ionization modes.

The MS was operated in 2 GHz extended dynamic range mode, and collision energies of 0 and 25 eV were applied. Chromatographic separation was achieved using an ACE 5 C18-PFP column (2.1 mm × 50 mm, 5 µm). Data acquisition and analysis were performed using Agilent MassHunter Software (version B06.00, Agilent Technologies, Inc., Santa Clara, CA, USA), and metabolite identification was supported by the METLIN Metabolite Database. Detailed HPLC and MS parameters are provided in Table 12.

### 4.3. Sample Preparation

An accurately weighed portion of the prepared extract (65.0 ± 0.1 mg) was dissolved in 10.0 mL of methanol. The solution was vortex-mixed for 2 min and subsequently sonicated in an ultrasonic bath for 30 min. An aliquot of 200 µL of this solution was further diluted with 800 µL of methanol prior to LC–MS analysis.

### 4.4. Calibration Solutions

Reference standards of Bergapten, Luteolin, and Biochanin A were accurately weighed (10.0 ± 0.1 mg each) and dissolved in methanol to prepare stock solutions at a concentration of 1 mg/mL.

Calibration solutions for all three compounds were prepared at seven concentration levels (1, 2.5, 5, 10, 25, 50, and 100 µg/mL) by appropriate serial dilutions. All dilution steps were carried out using methanol.

### 4.5. Animals

A total of 48 male Wistar albino rats aged 6–8 weeks, an age range considered adolescent in rats, were used in this study. Animals were obtained from the Gazi University Laboratory Animals Breeding and Experimental Research Center (GÜDAM), Ankara, Türkiye. All animals were healthy at the time of procurement, free of any genetic mutation/modification (wild-type), and had not undergone any prior experimental procedures. All experimental procedures were approved by the Gazi University Local Ethics Committee for Animal Experiments (approval no. G.Ü.ET-20.045). All animals were housed in standard cages under a 12 h light/dark cycle at 21–24 °C and 50 ± 5% relative humidity, with free access to a standard pellet diet and water. Animals were randomly allocated into six groups, each containing eight animals: Group Intact, Group Sham, Group Torsion–Detorsion (T/D), Group T/D + 125, Group T/D + 250, and Group T/D + 500. The number of animals per group was determined based on previously published studies on rats using comparable group sizes that evaluated natural or pharmacological protective agents for fertility-related parameters and testicular injury [59,60].

### 4.6. Induction of Testicular Torsion/Detorsion Model and Treatment

All procedures for animal experiments were conducted in accordance with the ARRIVE guidelines. All surgical procedures and extract administration were performed in the Laboratory Animals Breeding and Experimental Research Unit of Gazi University Faculty of Pharmacy. All rats underwent a 7-day acclimatization period prior to the initiation of the experimental procedures. No surgical procedures were performed on animals in the intact group, and these animals were maintained throughout the 56-day study period. All rats in the remaining groups were anesthetized with xylazine hydrochloride (8 mg/kg, i.p.; XylazinBio^®^, 2%; Interhas, Ankara, Türkiye) and ketamine hydrochloride (75 mg/kg, i.p.; Ketasol^®^, 10%; Interhas, Türkiye) before the surgical procedure. The left testis and spermatic cord of the animals in the sham group were exposed through a left scrotal incision, and then replaced in the scrotum following fixation. After the testis was fixed to the internal spermatic fascia, the scrotum was closed. The animals in the sham group were maintained until the day of sacrifice. The left testis and spermatic cord of the rats in the T/D, T/D + 125, T/D + 250, and T/D + 500 groups were exposed through a left scrotal incision, twisted 720° clockwise, and fixed to the internal spermatic fascia by suturing to establish an experimental testicular torsion model. After 60 min of torsion, the suture was removed, and detorsion was ensured. The left testis and spermatic cord were replaced in the scrotum in their anatomical positions and fixed to the internal spermatic fascia to prevent recurrent torsion, and then the scrotum was sutured. During the 56-day reperfusion period, animals in the intact, sham and T/D groups received the extract vehicle (0.5% aqueous carboxymethyl cellulose (CMC) solution) orally once daily, while animals in the T/D + 125, T/D + 250, and T/D + 500 groups received *F. carica* leaf extract orally at doses of 125, 250, and 500 mg/kg/day, respectively, prepared in the same vehicle. Daily administration of *F. carica* leaf extract was continued throughout the experiment. Selection of the doses was based on previous studies demonstrating the efficacy of comparable dose ranges for oral administration of *F. carica* leaf extract in rat/mouse models [22,25,28,29,30,38,61,62]. Previous studies evaluated oral *F. carica* leaf extract doses ranging from 100 mg/kg to 800 mg/kg in various mouse and rat experimental models [25,28,29,61,62]. In one of these studies by Fahmy and colleagues, the effectiveness of oral *F. carica* leaf extract at doses of 200, 400, and 600 mg/kg in preventing genotoxicity and testicular damage was investigated in a mouse model of cisplatin-induced toxicity [28].

The 56-day reperfusion period was selected to encompass approximately one complete spermatogenic cycle in rats, thereby allowing assessment of the longer-term effects of torsion/detorsion and *F. carica* leaf extract treatment on testicular structure and sperm parameters [63,64].

To minimize bias, the investigators who performed the surgical procedures were different from those who conducted the biochemical, histological, and immunohistochemical evaluations, and all evaluators were blinded to group allocation. Tissue specimens were coded with numeric identifiers only, without reference to group names, throughout biochemical, histological, and immunohistochemical analyses.

### 4.7. Sacrification and Sample Collection

At the end of the 56-day experimental period, all animals (except two animals, one from Group Sham and one from Group T/D, which died after surgical intervention) were anesthetized with xylazine hydrochloride (8 mg/kg, i.p.; XylazinBio^®^, 2%; Interhas, Türkiye) and ketamine hydrochloride (75 mg/kg, i.p.; Ketasol^®^, 10%; Interhas, Türkiye). Cardiac blood samples were collected from the rats under anesthesia to measure serum levels of Total Antioxidant Status (TAS), Total Oxidant Status (TOS), and Paraoxonase-1 (PON-1) activity, as well as serum levels of FSH, LH, and testosterone. The bilateral epididymides were used for sperm collection. Following weighing, each testis was immediately sectioned in half. One-half of both right and left testes were placed in liquid nitrogen and stored at −80 °C until analysis of tissue TAS and TOS levels and PON-1 activity. The other halves were immersed and fixed in 10% buffered formalin for histological and immunohistochemical analyses.

### 4.8. Evaluation of Sperm Counts and Motility

After dissection of the epididymides, spermatozoa collected bilaterally were transferred to the insemination medium (Sil-Select Plus^TM^, FertiPro, Beernem, Belgium). Afterward, sperm count and motility were evaluated under a light microscope using a Makler counting chamber.

### 4.9. Measurement of Serum Levels of FSH, LH, and Testosterone

Serum levels of FSH (E-EL-R0391, Elabscience, Houston, TX, USA), LH (E-EL-R0026, Elabscience, Houston, TX, USA), and testosterone (E-EL-0155, Elabscience, Houston, TX, USA) were measured by using ELISA kits. The manufacturer’s instructions were followed for all assay procedures.

### 4.10. Measurement of Serum and Tissue Levels of TAS, TOS, and PON-1 Activity

TAS and TOS levels and PON-1 activity were measured in serum and testicular tissue samples using commercially available colorimetric assay kits (TAS: RL0017; TOS: RL0024; PON-1: RL0031; Rel Assay Diagnostics, Gaziantep, Türkiye). Assay procedures were carried out according to the manufacturer’s instructions. Additionally, the ratio of TOS to TAS was used to calculate the Oxidative Stress Index (OSI) [65].

### 4.11. Histological and Immunohistochemical Examination

Seven µm thick serial sections of the testicular samples were cut from paraffin blocks using a microtome (Leica RM2245, Leica Biosystems Nussloch GmbH, Nussloch, Germany). Paraffin sections stained with hematoxylin and eosin (H&E) were examined under different magnifications using a light microscope (Leica DM 4000B, Leica Microsystems Wetzlar GmbH, Wetzlar, Germany) to evaluate the spermatogenesis through the Johnsen’s score [66]. For this purpose, 100 seminiferous tubule profiles per section were evaluated. Briefly, each seminiferous tubule cross-section is assigned a score from 1 to 10 based on the presence and organization of the spermatogenic cell layers: a score of 10 reflects complete spermatogenesis with a full complement of germ cell types and numerous mature spermatozoa, progressively lower scores reflect increasing degrees of germ cell layer depletion and disorganization, and a score of one reflects the complete absence of germinal and Sertoli cells within the tubule [66,67]. Then, a mean score was determined for each testis to compare the groups. Images of H&E-stained sections were captured under 100×, 200×, and 400× magnification using LAS V4.12 software (Leica Microsystems (Switzerland) Ltd., Heerbrugg, Switzerland).

Additionally, paraffin sections were immunohistochemically stained with antibodies against tumor necrosis factor-α (TNF-α) (1:100) (bs-2081R, Bioss-USA, Woburn, MA, USA) and interleukin 10 (IL-10) (1:100) (bs-0698R, Bioss-USA, Woburn, MA, USA) to evaluate the inflammatory and anti-inflammatory processes, or against Bcl-2-associated X protein (BAX) (1:100) (bs-0127R, Bioss-USA, Woburn, MA, USA), B-cell lymphoma 2 (BCL-2) (1:100) (bs-0032R, Bioss-USA, Woburn, MA, USA) and caspase-3 (CASP3) (1:200) (bs-0081R, Bioss-USA, Woburn, MA, USA) to evaluate apoptosis. For immunohistochemical staining, the UltraVision Detection System Anti-Polyvalent HRP Kit (TP-125-HL, Thermo Fisher Scientific, Waltham, MA, USA) was utilized. To visualize immunoreactivity, the 3,3’-diaminobenzidine (DAB) chromogen–substrate complex (TA-125-HDX, Thermo Fisher Scientific, USA) was used. Immunohistochemical procedures on sections from all animals, stained with the same antibody, were performed simultaneously under the same experimental conditions to ensure consistency. To that end, paraffin tissue sections were rehydrated and placed in citrate buffer (pH 6.0) for heat-induced epitope retrieval. After the sections were cooled, endogenous peroxidase activity was blocked using hydrogen peroxide. Then, the protein block solution was applied to the sections to prevent nonspecific antibody binding, followed by overnight incubation with primary antibodies at +4 °C. Later, sections were incubated with biotinylated secondary antibody. After the sections were incubated with streptavidin–peroxidase enzyme complex, the immunoreactivity was visualized using DAB chromogen–substrate complex, with an optimized concentration and duration, and applied identically across all sections to ensure comparable staining intensity. Negative control sections, processed without primary antibody, were included for each staining set.

To assess the TNF-α, IL-10, BAX, BCL-2, and CASP3 immunostaining intensities, all immunostained sections were examined under different magnifications using a light microscope (Leica DM 4000B, Germany), and micrographs of six non-overlapping fields from each section were captured at 200× magnification under the same microscopic settings using LAS V4.12 software. Immunostaining intensities for each marker were quantified in these micrographs using ImageJ software (1.54p; National Institutes of Health, Bethesda, MD, USA) and expressed as “% area” [68]. To compare immunostaining intensity, the brown DAB chromogen signal was isolated via color deconvolution, and a uniform threshold was adjusted based on images of negative control sections and applied across all specimens to distinguish positively stained (brown) areas from the background. Higher-magnification (400×) images of immunostained specimens were also presented alongside the representative 200× magnification images.

### 4.12. Statistical Analyses

Statistical analyses were conducted using SPSS software (version 26.0) using the statistical tests described below, and *p* < 0.05 was considered statistically significant. Ipsilateral and contralateral parameters obtained from the same animals were analyzed as independent samples. The Kolmogorov–Smirnov test was used for continuous variables to assess normality. Normally distributed data were expressed as mean ± standard deviation (SD), while categorical data were expressed as *n* (%); ANOVA effect size (Eta-squared), and 95% confidence intervals (CI) were reported for relevant estimates. To evaluate differences among groups, normally distributed data were analyzed using the independent samples ANOVA test. In the event of a significant overall difference, the Bonferroni post hoc test was utilized for pairwise comparisons. Sperm motility was compared using χ2 test and Fisher’s exact test.

## 5. Conclusions

In conclusion, administration of *F. carica* leaf extract was associated with attenuation of oxidative, inflammatory, and apoptotic markers following testicular torsion–detorsion. As no direct molecular pathway analyses, such as evaluation of NF-κB, Nrf2, MAPK, or mitochondrial apoptotic signaling, were performed, these findings remain to be comprehensively investigated in further studies to reveal the exact mechanisms of effect by mechanistic analysis. Although administration of *F. carica* leaf extract improved histological parameters of germ cell maturity and, to some extent, the sperm counts, the explanation for the dose-dependent elevation in serum FSH warrants further molecular and endocrine assessments. These results collectively provide a preliminary pharmacological basis for considering *F. carica* leaf extract as a candidate adjunctive agent in testicular ischemia–reperfusion injury; however, because these findings derive from a relatively small, standardized animal model that differs substantially from the variable clinical presentation of human testicular torsion, further pharmacological, toxicological, pharmacokinetic, and translational studies—including evaluation of optimal dosage, sperm genetic quality, and reproductive outcomes—are needed before *F. carica* leaf extract can be considered for potential clinical application.

## Figures and Tables

**Figure 1:**
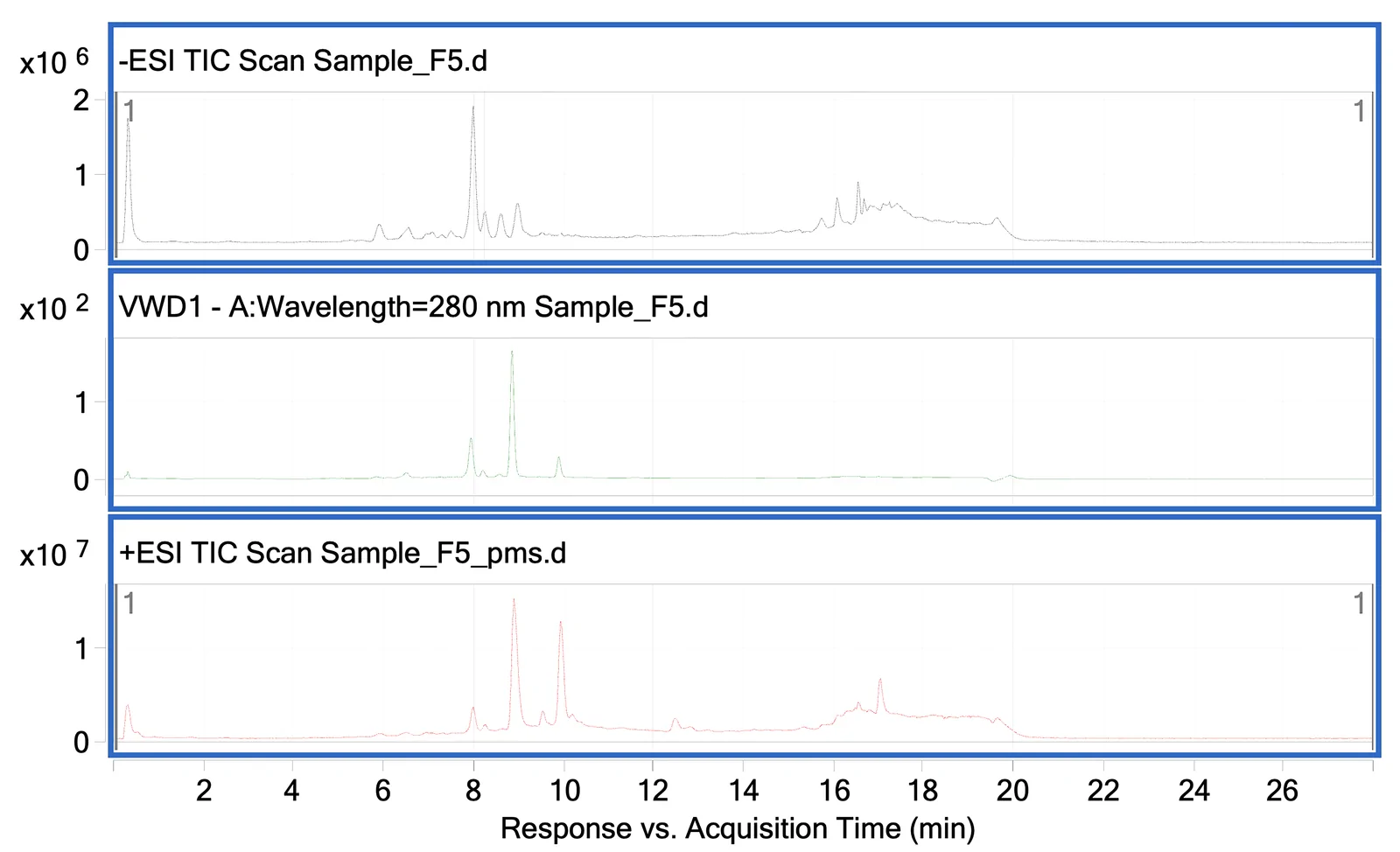
MS negative ion mode total ion chromatogram (TIC) of the sample (top), UV chromatogram at 280 nm (middle), and MS positive ion mode total ion chromatogram (TIC) (bottom).

**Figure 2:**
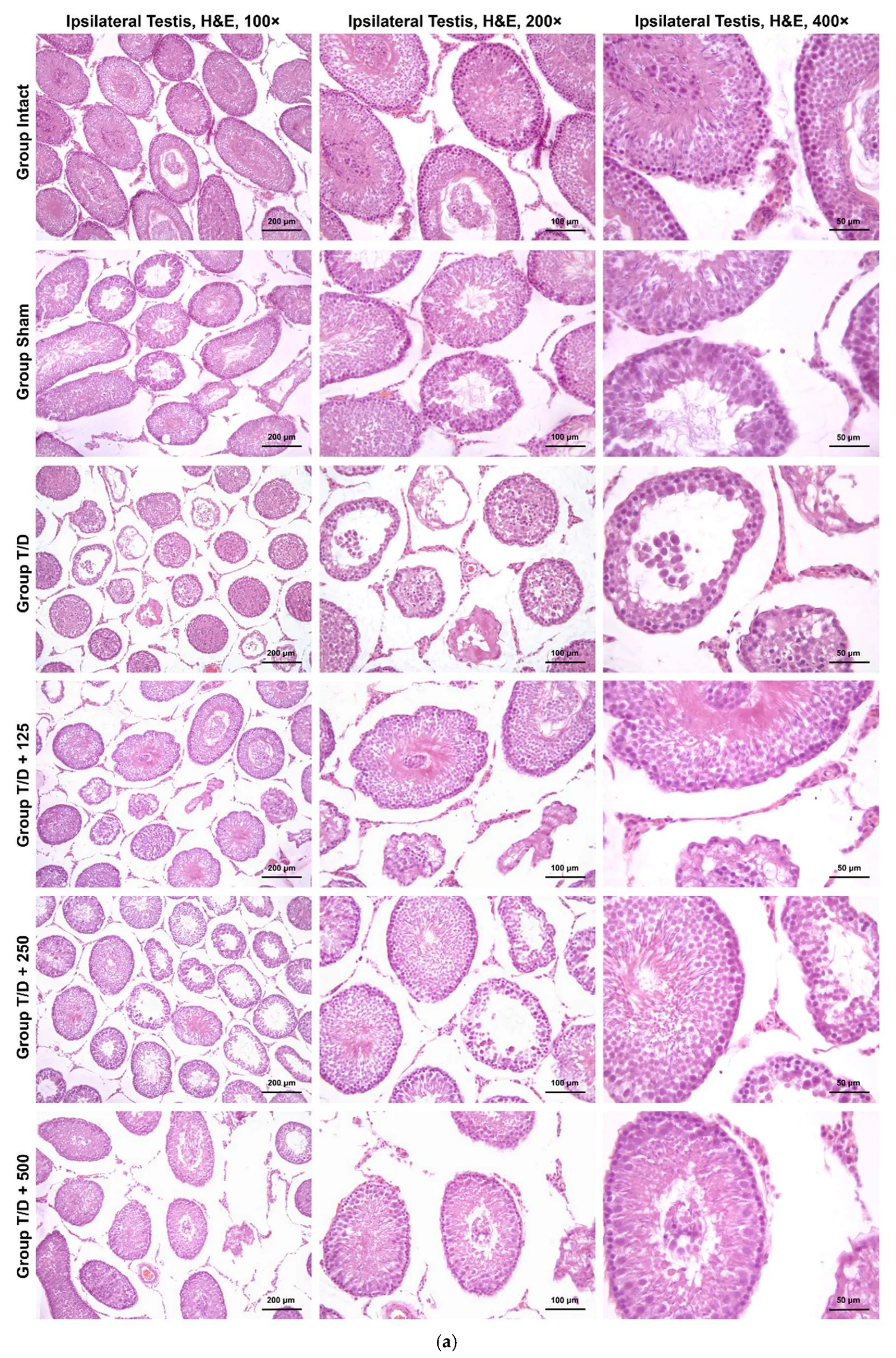

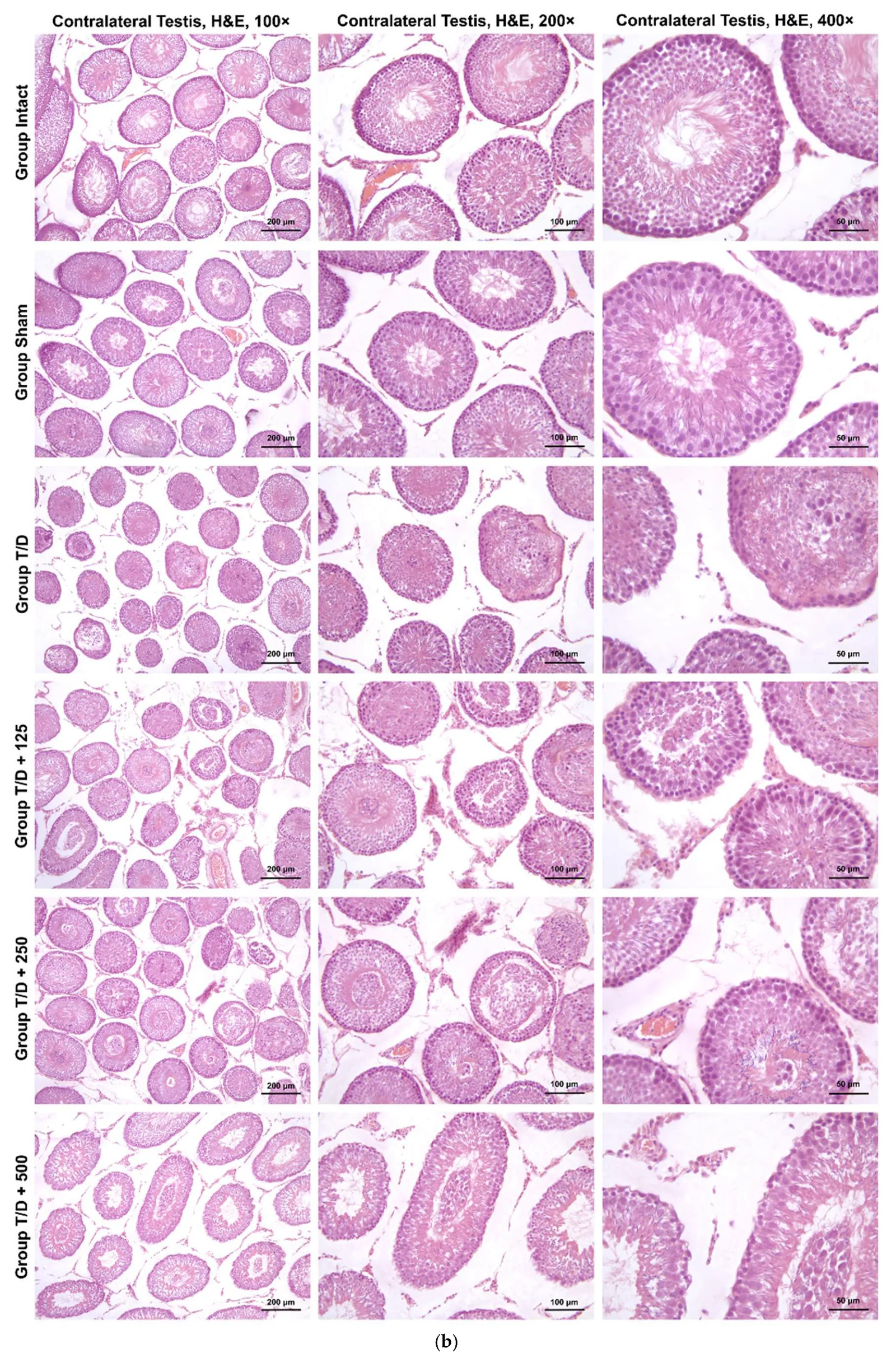
(**a**) Micrographs showing H&E-stained sections of the ipsilateral testis specimens. (**b**) Micrographs showing H&E-stained sections of the contralateral testis specimens. The T/D group underwent torsion–detorsion (T/D) only, whereas the T/D + 125, T/D + 250, and T/D + 500 groups received 125, 250, and 500 mg/kg *F. carica* leaf extract after T/D, respectively. H&E, hematoxylin and eosin staining. Magnification: 100×, 200×, and 400×.

**Figure 3:**
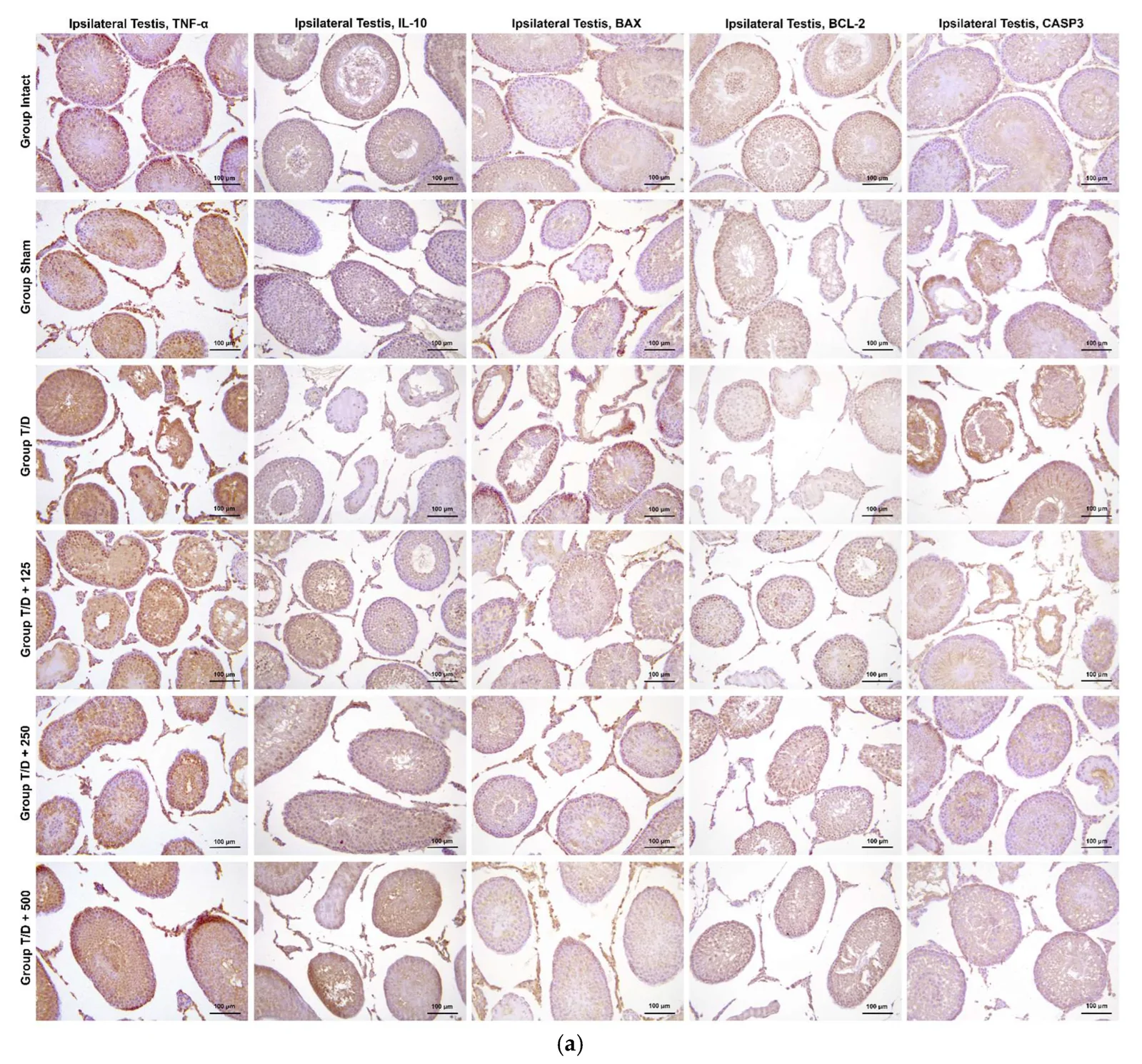

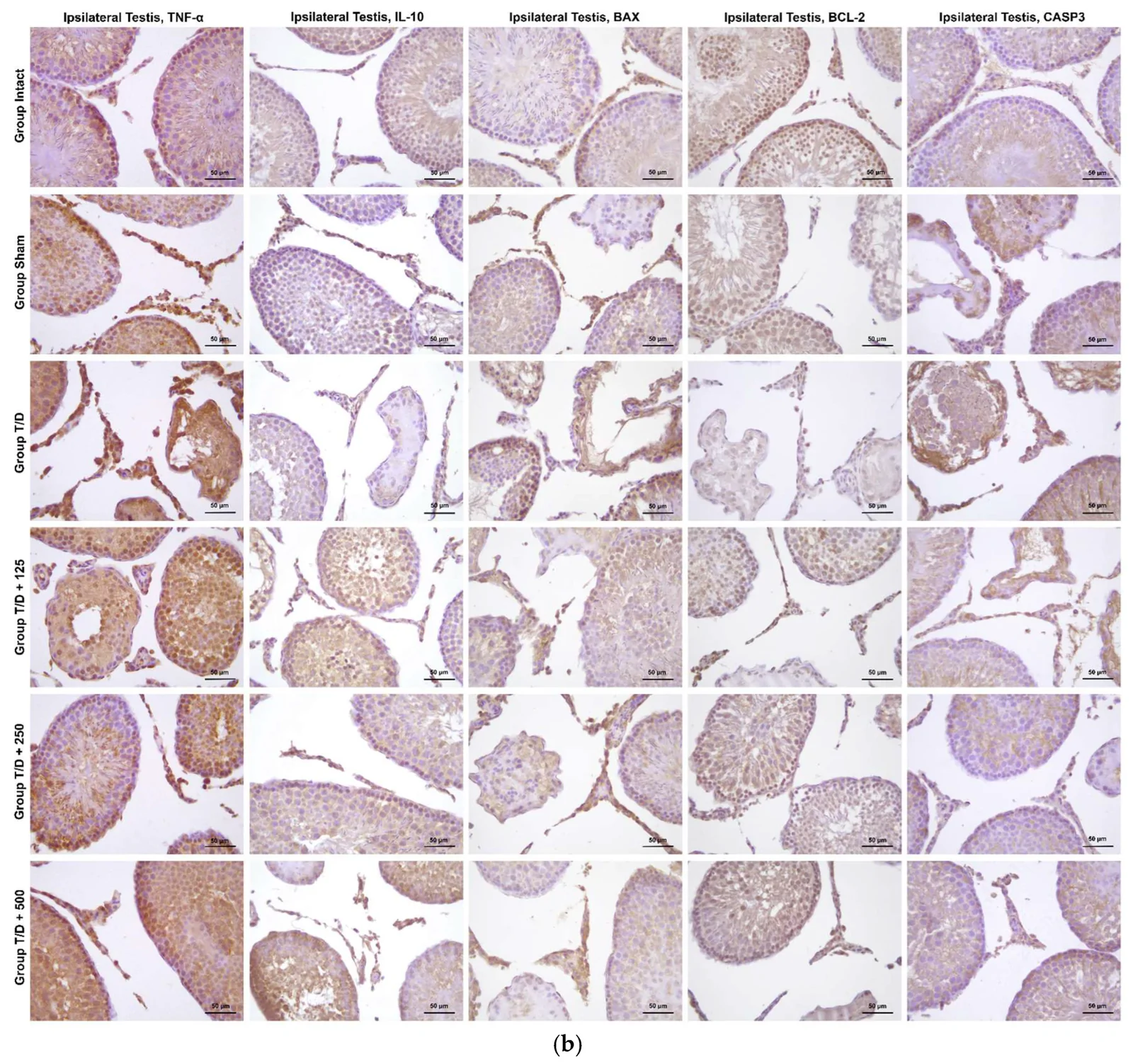
(**a**) Lower magnification (200×) micrographs showing TNF-α, IL-10, BAX, BCL-2, and CASP3- immunostained sections of the ipsilateral testis specimens. (**b**) Higher magnification (400×) micrographs showing TNF-α, IL-10, BAX, BCL-2, and CASP3- immunostained sections of the ipsilateral testis specimens. Tumor necrosis factor-α (TNF-α), interleukin-10 (IL-10), Bcl-2-associated X protein (BAX), B-cell lymphoma-2 (BCL-2), and caspase-3 (CASP3). T/D, T/D + 125, T/D + 250, and T/D + 500 groups are torsion-detorsion (T/D) applied-only, and 125 mg/kg, 250 mg/kg, and 500 mg/kg *F. carica* leaf extract-administered groups after T/D, respectively. Positive immunoreactivity for each marker was visualized using DAB (brown) staining.

**Figure 4:**
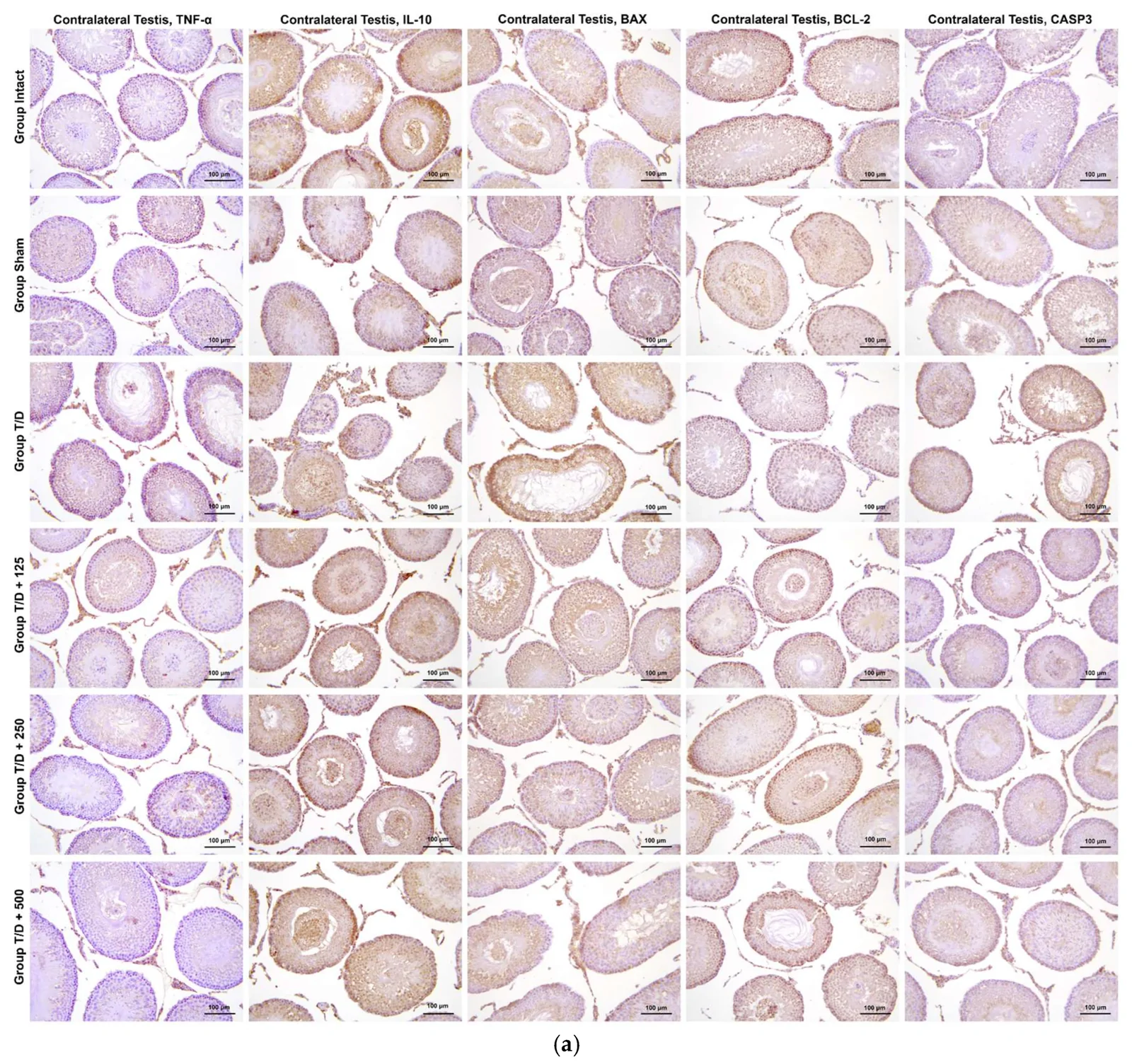

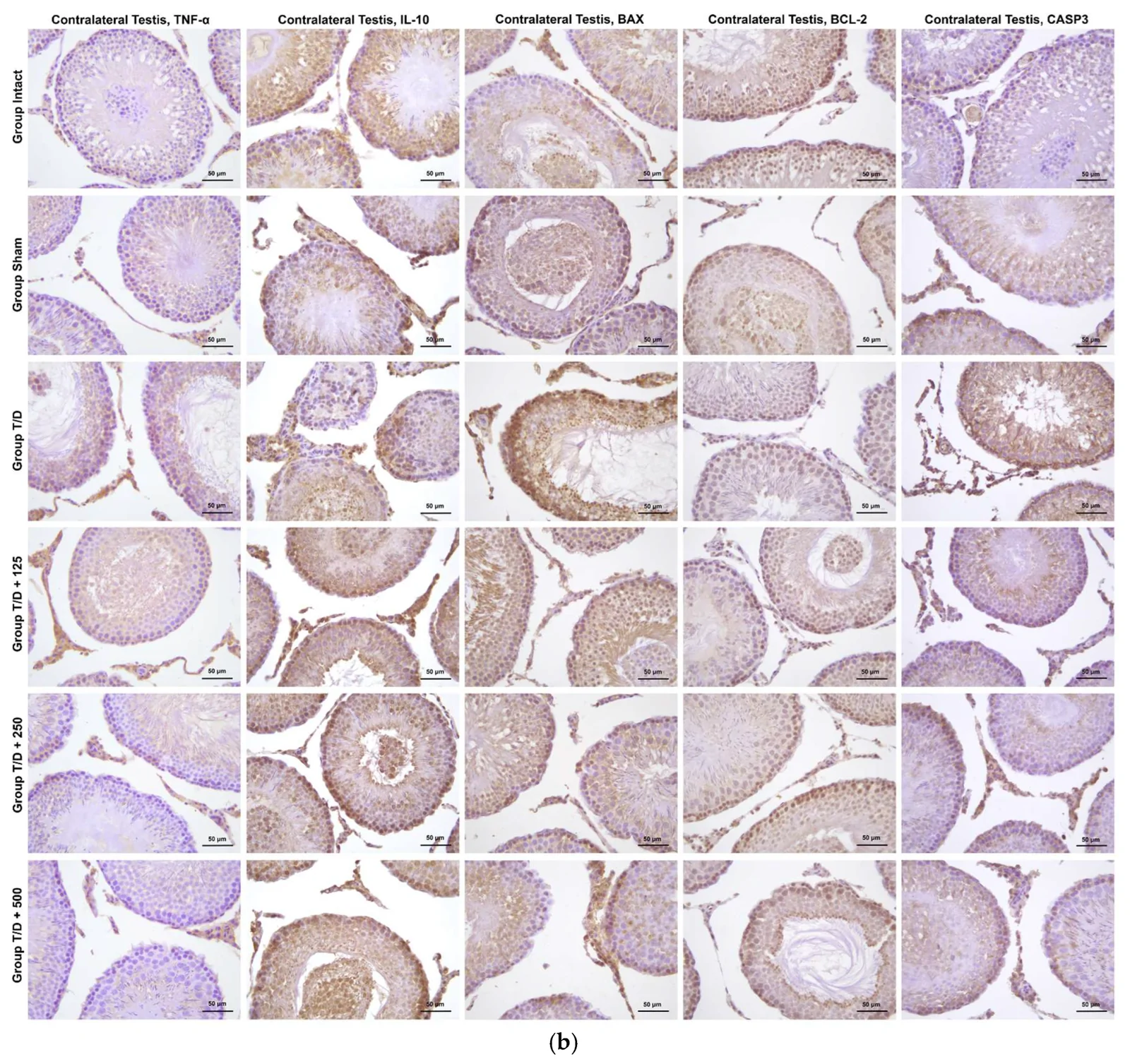
(**a**) Lower magnification (200×) micrographs showing TNF-α, IL-10, BAX, BCL-2, and CASP3- immunostained sections of the contralateral testis specimens. (**b**) Higher magnification (400×) micrographs showing TNF-α, IL-10, BAX, BCL-2, and CASP3- immunostained sections of the contralateral testis specimens. Tumor necrosis factor-α (TNF-α), interleukin-10 (IL-10), Bcl-2-associated X protein (BAX), B-cell lymphoma-2 (BCL-2), and caspase-3 (CASP3). The T/D group underwent torsion-detorsion (T/D) only, whereas the T/D + 125, T/D + 250, and T/D + 500 groups received 125, 250, and 500 mg/kg *F. carica* leaf extract after T/D, respectively. Positive immunoreactivity for each marker was visualized using DAB (brown) staining.

**Table 1:** Quantitative results of Bergapten, Luteolin, and Biochanin A.

Compound	RT (min)	Amount (mg/g)	LOQ (mg/g)
rgapten	9.90	13.2 ± 0.30	0.15
Luteolin	10.06	<LOQ	0.15
Biochanin A	12.34	<LOQ	0.15

**Table 2:** LC–MS Q-TOF characteristics of the compounds identified in *F. carica* leaf extract.

Compound	RT (min)	Molecular Formula	[M + H]/[M − H] Ion	Fragment Ions	UV Peak Area
Psoralen acid glucoside	7.96	C_17_H_18_O_9_	(M − H) 365.0849	203.0348	374.5 (37%)
Unidentified	8.22	–	(M − H) 395.0942	233.04	58.58 (5.8%)
Fellodenol A	8.58	C_11_H_14_O_4_	(M − H) 161.0607, 187.0394, 205.0498	—	30.79 (3.1%)
Psoralen	8.86	C_11_H_6_O_3_	(M + H) 187.0388	131.0573, 159.0470, 115.0643	1013.51 (100%)
Bergapten	9.90	C_12_H_8_O_4_	(M − H) 217.0436	202.0228, 174.0327	162.74 (16.1%)

**Table 3:** Results of body and testicular weight measurements [mean ± SD].

	Group Intact (*n* = 8)	Group Sham (*n* = 7)	Group T/D (*n* = 7)	Group T/D + 125 (*n* = 8)	Group T/D + 250 (*n* = 8)	Group T/D + 500 (*n* = 8)	ANOVA Effect Size	*p*
Initial body weight (g)	193.38 ± 19.87	206.71 ± 16.55	195.14 ± 17.01	198.00 ± 16.47	187.63 ± 19.18	201.25 ± 18.19	0.117	0.420
Final body weight (g)	300.13 ± 33.07	308.57 ± 25.24	287.00 ± 25.96	289.63 ± 42.08	285.13 ± 21.44	303.50 ± 31.31	0.085	0.597
Ipsilateral testis weight (g)	2.69 ± 0.34	2.15 ± 0.85 *	1.51 ± 0.32 *, &	1.41 ± 0.26 *, &	1.47 ± 0.36 *, &	1.39 ± 0.29 *, &	0.593	<0.001
Relative ipsilateral testis weight	0.91 ± 0.16	0.69 ± 0.23 *	0.53 ± 0.14 *, &	0.49 ± 0.09 *, &	0.52 ± 0.12 *, &	0.50 ± 0.04 *, &	0.574	<0.001
Contralateral testis weight (g)	2.58 ± 0.31	2.57 ± 0.33	2.50 ± 0.28	2.50 ± 0.15	2.37 ± 0.22	2.52 ± 0.13	0.090	0.562
Relative contralateral testis weight	0.87 ± 0.12	0.83 ± 0.06	0.87 ± 0.11	0.87 ± 0.09	0.83 ± 0.04	0.84 ± 0.09	0.054	0.804

T/D, T/D + 125, T/D + 250, and T/D + 500 groups are torsion–detorsion (T/D) applied-only, and 125 mg/kg, 250 mg/kg, and 500 mg/kg *F. carica* leaf extract-administered groups after T/D, respectively. * *p* < 0.05: Compared to the Group Intact; & *p* < 0.05: Compared with the Group Sham.

**Table 4:** Sperm counts [mean ± SD] and sperm motility (*n* (%)).

	Group Intact (*n* = 8)	Group Sham (*n* = 7)	Group T/D (*n* = 7)	Group T/D + 125 (*n* = 8)	Group T/D + 250 (*n* = 8)	Group T/D + 500 (*n* = 8)	ANOVA Effect Size	*p*
Ipsilateral epididymal sperm count (×10^6^)	45.38 ± 5.97	28.29 ± 12.71 *	19.14 ± 16.95 *	17.63 ± 9.86 *	17.88 ± 8.18 *	15.38 ± 8.02 *, &	0.532	<0.001
Contralateral epididymal sperm count (×10^6^)	47.00 ± 7.23	39.14 ± 5.87 *	51.57 ± 3.91 &	50.75 ± 7.09 &	58.38 ± 5.76 *, &, †, #	51.38 ± 8.30 &	0.464	<0.001
Total sperm count (×10^6^)	92.38 ± 11.16	67.43 ± 12.71 *	70.71 ± 17.01 *	68.38 ± 13.14 *	76.25 ± 8.22 *	66.75 ± 14.57 *	0.360	0.002
Ipsilateral epididymal sperm motility (positive/negative)	8(100)/0(0)	7(100)/0(0)	4(57.1)/3(42.9)*, &	2(25)/6(75) *, &	3(37.5)/5(62.5) *, &	5(62.5)/3(37.5)	-	χ2 = 20.874 <0.001
Contralateral epididymal sperm motility (positive/negative)	8(100)/0(0)	7(100)/0(0)	7(100)/0(0)	8(100)/0(0)	8(100)/0(0)	8(100)/0(0)	-	-

T/D, T/D + 125, T/D + 250, and T/D + 500 groups are torsion–detorsion (T/D) applied-only, and 125 mg/kg, 250 mg/kg, and 500 mg/kg *F. carica* leaf extract-administered groups after T/D, respectively. * *p* < 0.05: Compared to the Group Intact; & *p* < 0.05: Compared to the Group Sham; † *p* < 0.05: Compared to the Group T/D; # *p* < 0.05: Compared to the Group T/D + 125.

**Table 5:** Serum FSH, LH, and testosterone levels [mean ± SD].

	Group Intact (*n* = 8)	Group Sham (*n* = 7)	Group T/D (*n* = 7)	Group T/D + 125 (*n* = 8)	Group T/D + 250 (*n* = 8)	Group T/D + 500 (*n* = 8)	ANOVA Effect Size	*p*
FSH (ng/mL)	58.40 ± 18.70	106.38 ± 28.91 *	73.89 ± 29.32	104.63 ± 26.01 *	148.15 ± 53.52 *, &, †, #	194.63 ± 35.16 *, &, †, #, %	0.681	<0.001
LH (mIU/mL)	8.02 ± 1.71	11.30 ± 3.88	9.18 ± 1.70	8.16 ± 4.04	9.45 ± 2.12	7.08 ± 1.83	0.212	0.079
Testosterone (ng/mL)	4.91 ± 1.11	4.79 ± 2.12	5.77 ± 0.98	5.38 ± 2.06	5.96 ± 2.38	3.34 ± 1.58	0.215	0.075

Follicle-stimulating hormone (FSH), luteinizing hormone (LH). T/D, T/D + 125, T/D + 250, and T/D + 500 groups are torsion–detorsion (T/D) applied-only, and 125 mg/kg, 250 mg/kg, and 500 mg/kg *F. carica* leaf extract-administered groups after T/D, respectively. * *p* < 0.05: Compared to the Group Intact; & *p* < 0.05: Compared to the Group Sham; † *p* < 0.05: Compared to the Group T/D; # *p* < 0.05: Compared to the Group T/D + 125; % *p* < 0.05: Compared to the Group T/D + 250.

**Table 6:** Serum TAS and TOS levels, OSI values, and PON-1 activity [mean ± SD].

	Group Intact (*n* = 8)	Group Sham (*n* = 7)	Group T/D (*n* = 7)	Group T/D + 125 (*n* = 8)	Group T/D + 250 (*n* = 8)	Group T/D + 500 (*n* = 8)	ANOVA Effect Size	*p*
TAS (mmol/L)	1.41 ± 0.15	1.41 ± 0.09	1.42 ± 0.13	1.47 ± 0.13	1.37 ± 0.03	1.30 ± 0.10	0.212	0.080
TOS (µmol/L)	3.76 ± 6.11	2.81 ± 2.31	7.39 ± 6.86	2.39 ± 2.67	2.48 ± 2.07	3.57 ± 2.92	0.150	0.240
OSI	0.26 ± 0.42	0.20 ± 0.16	0.51 ± 0.47	0.16 ± 0.18	0.18 ± 0.15	0.27 ± 0.20	0.147	0.253
PON-1 (U/L)	626.75 ± 72.03	697.43 ± 47.51	608.71 ± 107.97	616.25 ± 111.17	667.00 ± 108.09	657.25 ± 71.45	0.117	0.398

Total Antioxidant Status (TAS), Total Oxidant Status (TOS), Oxidative Stress Index (OSI), and Paraoxonase-1 (PON-1). T/D, T/D + 125, T/D + 250, and T/D + 500 groups are torsion–detorsion (T/D) applied-only, and 125 mg/kg, 250 mg/kg, and 500 mg/kg *F. carica* leaf extract-administered groups after T/D, respectively.

**Table 7:** TAS and TOS levels, OSI values, and PON-1 activity in ipsilateral testis specimens [mean ± SD].

	Group Intact (*n* = 8)	Group Sham (*n* = 7)	Group T/D (*n* = 7)	Group T/D + 125 (*n* = 8)	Group T/D + 250 (*n* = 8)	Group T/D + 500 (*n* = 8)	ANOVA Effect Size	*p*
TAS (mmol/L)	2.06 ± 0.11	1.80 ± 0.38 *	1.40 ± 0.09 *, &	1.54 ± 0.20 *, &	1.48 ± 0.15 *, &	1.54 ± 0.15 *, &	0.587	<0.001
TOS (µmol/L)	8.34 ± 1.59	9.07 ± 1.57	9.46 ± 2.58	8.82 ± 1.83	8.00 ± 2.75	4.73 ± 0.85 *, &, †, #, %	0.427	<0.001
OSI	0.41 ± 0.08	0.52 ± 0.13	0.67 ± 0.15 *, &	0.58 ± 0.14 *	0.54 ± 0.18	0.32 ± 0.06 &, †, #, %	0.334	<0.001
PON-1 (U/L)	7.85 ± 1.11	10.36 ± 3.79	11.58 ± 4.21	10.38 ± 4.41	8.04 ± 1.84	8.67 ± 2.60	0.174	0.161

Total Antioxidant Status (TAS), Total Oxidant Status (TOS), Oxidative Stress Index (OSI), and Paraoxonase-1 (PON-1). T/D, T/D + 125, T/D + 250, and T/D + 500 groups are torsion–detorsion (T/D) applied-only, and 125 mg/kg, 250 mg/kg, and 500 mg/kg *F. carica* leaf extract-administered groups after T/D, respectively. * *p* < 0.05: Compared to the Group Intact; & *p* < 0.05: Compared to the Group Sham; † *p* < 0.05: Compared to the Group T/D; # *p* < 0.05: Compared to the Group T/D + 125; % *p* < 0.05: Compared to the Group T/D + 250.

**Table 8:** TAS and TOS levels, OSI values, and PON-1 activity in contralateral testis specimens [mean ± SD].

	Group Intact (*n* = 8)	Group Sham (*n* = 7)	Group T/D (*n* = 7)	Group T/D + 125 (*n* = 8)	Group T/D + 250 (*n* = 8)	Group T/D + 500 (*n* = 8)	ANOVA Effect Size	*p*
TAS (mmol/L)	2.12 ± 0.20	1.97 ± 0.18	1.39 ± 0.10 *, &	1.59 ± 0.19 *, &, †	1.64 ± 0.09 *, &, †	1.78 ± 0.21 *, &, †, #	0.694	<0.001
TOS (µmol/L)	7.16 ± 1.27	8.42 ± 1.00	9.50 ± 1.51 *	8.32 ± 1.70	7.60 ± 0.94	6.50 ± 1.66 &, †, #	0.354	0.003
OSI	0.34 ± 0.05	0.43 ± 0.05	0.68 ± 0.16 *, &	0.54 ± 0.16 *, †	0.47 ± 0.06 *, †	0.37 ± 0.07 †, #	0.568	<0.001
PON-1 (U/L)	9.12 ± 1.40	11.03 ± 7.68	10.33 ± 2.30	8.62 ± 2.42	8.78 ± 2.17	8.97 ± 2.08	0.064	0.740

Total Antioxidant Status (TAS), Total Oxidant Status (TOS), Oxidative Stress Index (OSI), and Paraoxonase-1 (PON-1). T/D, T/D + 125, T/D + 250, and T/D + 500 groups are torsion–detorsion (T/D) applied-only, and 125 mg/kg, 250 mg/kg, and 500 mg/kg *F. carica* leaf extract-administered groups after T/D, respectively. * *p* < 0.05: Compared to the Group Intact; & *p* < 0.05: Compared to the Group Sham; † *p* < 0.05: Compared to the Group T/D; # *p* < 0.05: Compared to the Group T/D + 125.

**Table 9:** Johnsen’s scores in ipsilateral and contralateral testis specimens [mean ± SD].

	Group Intact (*n* = 8)	Group Sham (*n* = 7)	Group T/D (*n* = 7)	Group T/D + 125 (*n* = 8)	Group T/D + 250 (*n* = 8)	Group T/D + 500 (*n* = 8)	ANOVA Effect Size	*p*
Ipsilateral testis Johnsen’s score	8.57 ± 0.09	7.83 ± 0.88	5.12 ± 1.12 *, &	5.60 ± 1.49 *, &	6.53 ± 1.10 *, &, †	6.73 ± 1.05 *, †, #	0.429	<0.001
Contralateral testis Johnsen’s score	8.73 ± 0.13	8.48 ± 0.10 *	8.46 ± 0.15 *	8.41 ± 0.18 *	8.31 ± 0.15 *	8.48 ± 0.11 *	0.508	<0.001

T/D, T/D + 125, T/D + 250, and T/D + 500 groups are torsion–detorsion (T/D) applied-only, and 125 mg/kg, 250 mg/kg, and 500 mg/kg *F. carica* leaf extract-administered groups after T/D, respectively. * *p* < 0.05: Compared to the Group Intact; & *p* < 0.05: Compared to the Group Sham; † *p* < 0.05: Compared to the Group T/D; # *p* < 0.05: Compared to the Group T/D + 125.

**Table 10:** TNF-α, IL-10, BAX, BCL-2, and CASP3 immunostaining intensities (% area) in ipsilateral testis specimens [mean ± SD].

	Group Intact (*n* = 8)	Group Sham (*n* = 7)	Group T/D (*n* = 7)	Group T/D + 125 (*n* = 8)	Group T/D + 250 (*n* = 8)	Group T/D + 500 (*n* = 8)	ANOVA Effect Size	*p*
TNF-α immunostaining intensity (% area)	13.91 ± 5.18	19.50 ± 7.42	28.35 ± 9.09 *, &	19.58 ± 5.68 †	19.34 ± 7.45 †	19.83 ± 5.70 †	0.299	0.012
IL-10 immunostaining intensity (% area)	13.48 ± 8.67	9.38 ± 5.64	4.86 ± 4.22 *	14.65 ± 8.60 †	18.86 ± 10.82 &, †	20.96 ± 10.32 &, †	0.312	0.009
BAX immunostaining intensity (% area)	6.05 ± 2.31	11.06 ± 1.19 *	16.07 ± 2.86 *, &	11.15 ± 6.19 *, †	11.46 ± 6.20 *, †	6.89 ± 3.33 &, †	0.492	<0.001
BCL-2 immunostaining intensity (% area)	20.03 ± 13.37	13.77 ± 6.33	4.24 ± 3.46 *, &	12.01 ± 8.45	19.58 ± 9.94 †	18.84 ± 13.86 †	0.252	0.035
CASP3 immunostaining intensity (% area)	5.36 ± 3.07	12.60 ± 4.34 *	18.97 ± 6.92 *, &	10.93 ± 3.48 *, †	9.19 ± 4.51 †	5.86 ± 3.21 &, †, #	0.548	<0.001

Tumor necrosis factor-α (TNF-α), interleukin 10 (IL-10), Bcl-2-associated X protein (BAX), B-cell lymphoma 2 (BCL-2), and caspase-3 (CASP3). T/D, T/D + 125, T/D + 250, and T/D + 500 groups are torsion–detorsion (T/D) applied-only, and 125 mg/kg, 250 mg/kg, and 500 mg/kg *F. carica* leaf extract-administered groups after T/D, respectively. * *p* < 0.05: Compared to the Group Intact; & *p* < 0.05: Compared to the Group Sham; † *p* < 0.05: Compared to the Group T/D; # *p* < 0.05: Compared to the Group T/D + 125.

**Table 11:** TNF-α, IL-10, BAX, BCL-2, and CASP3 immunostaining intensities (% area) in contralateral testis specimens [mean ± SD].

	Group Intact (*n* = 8)	Group Sham (*n* = 7)	Group T/D (*n* = 7)	Group T/D + 125 (*n* = 8)	Group T/D + 250 (*n* = 8)	Group T/D + 500 (*n* = 8)	ANOVA Effect Size	*p*
TNF-α immunostaining intensity (% area)	3.99 ± 1.73	5.85 ± 2.00	11.46 ± 3.41 *, &	5.98 ± 3.85 †	5.07 ± 1.27 †	3.03 ± 0.91 &, †, #	0.573	<0.001
IL-10 immunostaining intensity (% area)	25.35 ± 7.81	13.01 ± 4.26 *	7.59 ± 3.35 *	18.99 ± 11.19 †	19.47 ± 5.58 †	20.59 ± 9.93 †	0.377	0.002
BAX immunostaining intensity (% area)	6.91 ± 4.66	9.44 ± 3.64	20.63 ± 6.07 *, &	11.76 ± 3.34 *, †	10.74 ± 5.78 †	7.28 ± 2.31 †	0.533	<0.001
BCL-2 immunostaining intensity (% area)	15.55 ± 7.54	9.49 ± 5.65	4.74 ± 3.37 *	9.48 ± 4.97 *	12.53 ± 7.25 †	12.34 ± 3.84 †	0.280	0.018
CASP3 immunostaining intensity (% area)	5.23 ± 2.47	9.24 ± 5.87	15.62 ± 6.29 *, &	7.73 ± 3.06†	7.52 ± 2.25 †	6.91 ± 1.66 †	0.440	<0.001

Tumor necrosis factor-α (TNF-α), interleukin 10 (IL-10), Bcl-2-associated X protein (BAX), B-cell lymphoma 2 (BCL-2), and caspase-3 (CASP3). T/D, T/D + 125, T/D + 250, and T/D + 500 groups are torsion–detorsion (T/D) applied-only, and 125 mg/kg, 250 mg/kg, and 500 mg/kg *F. carica* leaf extract-administered groups after T/D, respectively. * *p* < 0.05: Compared to the Group Intact; & *p* < 0.05: Compared to the Group Sham; † *p* < 0.05: Compared to the Group T/D; # *p* < 0.05: Compared to the Group T/D + 125.

**Table 12:** HPLC and MS analytical conditions.

Parameter	Condition
Column	ACE 5 C18-PFP (2.1 mm × 50 mm, 5 µm)
Column oven temperature	35 °C
Injection volume	5 µL
Run time	28 min
Mobile phase A	Water containing 0.1% acetic acid
Mobile phase B	Methanol
Flow rate	0.75 mL/min
Gradient program	0 min—5% B2 min—5% B6 min—30% B8 min—50%—B14 min—70% B15 min—90% B18 min—90% B18.1 min—5% B
UV detection wavelength	280 nm
Ionization mode	Positive and negative
Drying gas temperature	200 °C
Drying gas flow (N_2_)	14 L/min
Nebulizer pressure	40 psi
Sheath gas temperature	350 °C
Sheath gas flow (N_2_)	11 L/min
Capillary voltage	2000 V (positive and negative modes)
Nozzle voltage	1000 V
Mass range	*m/z* 50–3200
Reference ions	*m/z* 966.000725 (negative mode) *m/z* 922.0098 (positive mode)

## Data Availability

All data generated or analyzed during this study are included in this published article.
